# Condensin II Subunit dCAP-D3 Restricts Retrotransposon Mobilization in *Drosophila* Somatic Cells

**DOI:** 10.1371/journal.pgen.1003879

**Published:** 2013-10-31

**Authors:** Andrew T. Schuster, Kavitha Sarvepalli, Eain A. Murphy, Michelle S. Longworth

**Affiliations:** Department of Molecular Genetics, Lerner Research Institute, Cleveland Clinic Foundation, Cleveland, Ohio, United States of America; Harvard Medical School, United States of America

## Abstract

Retrotransposon sequences are positioned throughout the genome of almost every eukaryote that has been sequenced. As mobilization of these elements can have detrimental effects on the transcriptional regulation and stability of an organism's genome, most organisms have evolved mechanisms to repress their movement. Here, we identify a novel role for the *Drosophila melanogaster* Condensin II subunit, dCAP-D3 in preventing the mobilization of retrotransposons located in somatic cell euchromatin. dCAP-D3 regulates transcription of euchromatic gene clusters which contain or are proximal to retrotransposon sequence. ChIP experiments demonstrate that dCAP-D3 binds to these loci and is important for maintaining a repressed chromatin structure within the boundaries of the retrotransposon and for repressing retrotransposon transcription. We show that dCAP-D3 prevents accumulation of double stranded DNA breaks within retrotransposon sequence, and decreased dCAP-D3 levels leads to a precise loss of retrotransposon sequence at some dCAP-D3 regulated gene clusters and a gain of sequence elsewhere in the genome. Homologous chromosomes exhibit high levels of pairing in *Drosophila* somatic cells, and our FISH analyses demonstrate that retrotransposon-containing euchromatic loci are regions which are actually less paired than euchromatic regions devoid of retrotransposon sequences. Decreased dCAP-D3 expression increases pairing of homologous retrotransposon-containing loci in tissue culture cells. We propose that the combined effects of dCAP-D3 deficiency on double strand break levels, chromatin structure, transcription and pairing at retrotransposon-containing loci may lead to 1) higher levels of homologous recombination between repeats flanking retrotransposons in dCAP-D3 deficient cells and 2) increased retrotransposition. These findings identify a novel role for the anti-pairing activities of dCAP-D3/Condensin II and uncover a new way in which dCAP-D3/Condensin II influences local chromatin structure to help maintain genome stability.

## Author Summary

Condensins are conserved complexes that are well known for their roles in promoting the efficient condensation of chromosomes during early mitosis. Previously, we have shown that the *Drosophila* Condensin II subunit, dCAP-D3, also functions to regulate transcription in somatic cells during the later stages of development. A significant number of dCAP-D3 regulated genes were found to be positioned very close to one another in clusters. In this study, we report that some of the most strongly regulated dCAP-D3 gene clusters are positioned near retrotransposons. Unexpectedly, we find that decreased dCAP-D3 expression results in a precise loss of retrotransposon sequence at these loci. Additionally, dCAP-D3 knockdown causes increased levels of double strand breaks within retrotransposon sequence, an opening of the chromatin in the region, increased retrotransposon transcription and a very significant increase in homologous pairing at the locus. Taken together, these results suggest that dCAP-D3/Condensin II functions to prevent recombination of retrotransposons between homologous chromosomes and possibly retrotransposition as well. This report identifies a novel function for Condensin II that may contribute to its role in genome organization.

## Introduction

Condensins are complexes which are well known for their roles in ensuring efficient global chromatin condensation during prophase of mitosis [1]–[4]. Two Condensin complexes, Condensin I and Condensin II are conserved in multicellular eukaryotes. Each complex contains SMC2 and SMC4 proteins which heterodimerize to form ATPases that act to constrain positive supercoils [5], [6]. Mammalian Condensin I and II differ in their non-SMC subunits. Condensin I contains the kleisin, CAP-H, and two HEAT repeat proteins, CAP-D2 and CAP-G. Condensin II contains the kleisin, CAP-H2, and two HEAT repeat proteins, CAP-D3 and CAP-G2 (a CAP-G2 homolog has not been discovered in *Drosophila*). The two Condensin complexes bind to chromosomes differently and possess functions independent of one another [4], [6]–[10].

Another important difference between Condensin I and Condensin II is that, in mammals, Condensin II is present in the nucleus throughout the cell cycle, whereas Condensin I remains in the cytoplasm until nuclear envelope breakdown occurs in mitosis. This suggests that Condensin II may possess unique functions outside of mitosis, and in recent years, several reports have identified non-mitotic roles for the Condensin II complex. In human cells undergoing premature chromatin condensation, Condensin II component CAP-G2 was recently shown to be necessary for sister chromatid resolution during S phase [11]. Murine Condensin II component CAP-G2 was shown to be play a role in the differentiation of erythrocytes [12]. Additionally, plant Condensin II components prevent accumulation of DNA damage induced by drugs which block S phase progression [13].

Several interesting non-mitotic functions for the *Drosophila* Condensin II complex have also recently been identified. dCAP-H2 and dCAP-D3 subunits were found to be necessary for chromosome territory formation in non-mitotic tissues [14]. *Drosophila* somatic cells exhibit high levels of homologous chromosome pairing throughout the cell cycle, and Condensin II subunits have been thoroughly characterized to act as “anti-pairing” proteins both at heterochromatic and euchromatic sequence [9], [14]–[17]. While the mechanisms and full implications of the Condensin II anti-pairing function is not fully understood, it has been linked to transcriptional regulation; dCAP-H2 has been shown to antagonize transvection and prevent the transcriptional regulation of one allele by physical association with the homologous allele [16]. Previously, we demonstrated that dCAP-D3 regulates a significant number of genes during the later stages of *Drosophila* development and in non-dividing tissues [8]. Many of these genes are positioned adjacent to one another in clusters which can span over 50 kb. This suggests that the mechanism by which dCAP-D3 regulates transcription can affect multiple genes at once and can operate over large distances. However, the exact mechanisms of how dCAP-D3/Condensin II mediates transcriptional regulation are unknown.

Here, we show that some of the most highly misregulated gene clusters in dCAP-D3 mutants are located proximal to retrotransposon sequences. Natural Transposable Elements have been studied extensively for their potential to increase genetic variation through their mobilization within genomes. Retrotransposons are a class of Natural Transposable Elements that can mobilize through transcription of their own encoded retrotransposase and an RNA intermediate. This leads to a new copy being made and inserted into a novel site within the genome, while the old copy remains in the original locus. Given that retrotransposons are present in multiple copies in an organism, they can also mobilize through homologous recombination with allelic or non-allelic sequences on homologous and/or non-homologous chromosomes [18]–[24]. Retrotransposons have often been described as “selfish elements” since, if left unchecked, they would be free to move in and out of a host genome, potentially causing genomic instability due to loss or gain of accompanying host genome sequence [25]. In fact, the LINE-1 element, a type of retroelement in humans which makes up 17% of the human genome, has been shown to induce double strand breaks and its de-repression is associated with tumor development [26]–[29].

In this work, we uncover a novel role for the Condensin II complex in the prevention of retrotransposon mobilization in *Drosophila* somatic cells. Transcript levels of retrotransposons and the genes proximal to retrotransposons are significantly affected following their mobilization in dCAP-D3 deficient cells. We show that dCAP-D3 prevents double strand break accumulation within retrotransposon sequence and prevents pairing of these regions between homologous chromosomes. We present a working model in which Condensin II regulation of local and global chromatin architecture might act to restrict homologous recombination of retrotransposons and/or prevent retrotransposition.

## Results

### Decreased levels of dCAP-D3/Condensin II result in local losses of retrotransposon sequence

**Figure 1:**
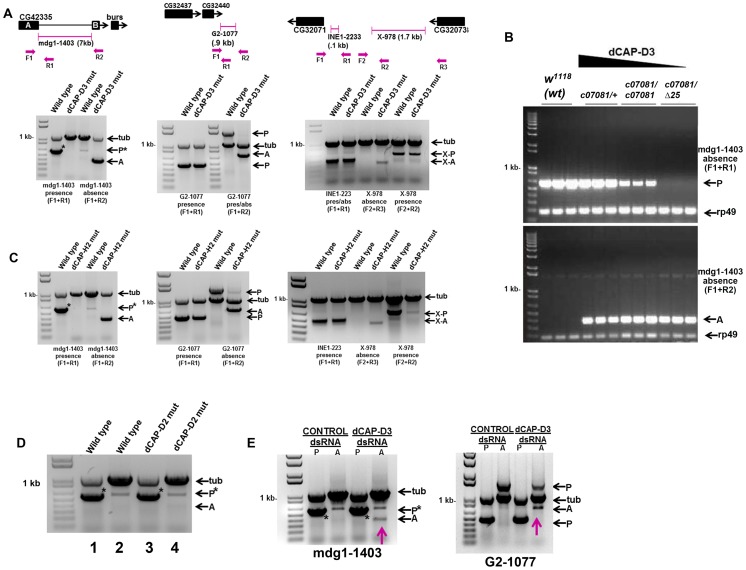
Decreased levels of dCAP-D3/Condensin II result in a local loss of retrotransposon sequence *in vivo* and *in vitro*. A) PCR performed on DNA from wild type (*w^1118^*) adults and dCAP-D3 transheterozygous mutant adults (d*Cap-D3^c07081^*d*Cap-D3^/Δ25^*) to detect whether three different retrotransposon sequences (*mdg1-1403, G2-1077, X-978*) were present (P) or absent (A) in dCAP-D3 regulated gene clusters indicates absence of the retrotransposon sequence only in *dCap-D3* mutants. In the diagram for each locus, transposon positions are illustrated with pink brackets. Single black boxes represent the entire coding sequence for each gene, except in the case of CG42335 where two black boxes are used to represent the coding sequence upstream and downstream of the *mdg1-1403* retrotransposon. Primers sets used are depicted in the diagrams above the gels and their sequences can be found in Materials and Methods. Tubulin23C (Tub) was used as a control for each reaction. In the PCRs performed on the *mdg1-1403* locus, an asterisk denotes the band for presence. The miscellaneous band seen in the wild type absence reaction was confirmed to be a mispriming event off of tubulin (data not shown). B) PCR performed to detect presence or absence of *mdg1-1403* in different *dCap-D3* mutant genotypes shows that events which cause local loss of retrotransposon sequence increase as dCAP-D3 expression levels decrease. PCR was performed in individual, female, wild type flies and flies expressing different *dCap-D3* mutant alleles. *dCap-D3* mutants were heterozygous or homozygous for a hypomorphic allele of *dCap-D3* (*c07081*) which expresses about 10% the levels of wild type protein [8], or transheterozygous for a combination of the hypomorphic allele and a deletion (*c07081/Δ25*). *rp49* was used as a control for each reaction. C) PCRs performed as described in (A) on DNA from flies expressing two mutant alleles of a second Condensin II subunit, *dCap-H2* (*dCap-H2^Z3-0019^/dCap-H2^Z3-^* ^5163^), demonstrates identical results seen for *dCap-D3* mutants. D) PCR for *mdg1-1403* presence or absence in Condensin I subunit, *dCap-D2*, heterozygous mutants *(dCap-D2^f03381^/+)* shows only presence of the retrotransposon. PCRs were performed on 1) wild type adults to test for presence, 2) wild type adults to test for absence, 3) *dCap-D2* mutants to test for presence and 4) *dCap-D2* mutants to test for absence. E) PCR for presence and absence of *mdg1-1403* (left) and *G2-1077* (right) in SG4 cells treated with control or dCAP-D3 dsRNAs for 6 days shows the appearance of an absence band (pink arrows) in dCAP-D3 dsRNA treated cells but not in control dsRNA treated cells.

Previously published microarray analyses of total RNA isolated from whole adult flies and whole larvae mutant for the Condensin II subunit, dCAP-D3, revealed that dCAP-D3 regulates a significant number of genes during these later stages of development. It was also determined that dCAP-D3 regulates clusters of genes at a frequency much higher than expected by random chance [8]. Upon comparison to the most current genome annotations for the *y[1]; cn[1] bw[1] sp[1] Drosophila* strain (deposited by Berkeley *Drosophila* Genome Project in Flybase.org), it was determined that some of the most highly misregulated dCAP-D3 target gene clusters were located within 5 kb of a retrotransposon. Since transposable element positioning is variable between strains, it was necessary to confirm that the annotated retrotransposon positions for *y[1]; cn[1] bw[1] sp[1]* were also correct for the *w^1118^* strain which was used as the wild type stock in our microarray experiments. dCAP-D3 mutant stocks were originally generated in a *w^1118^* background and prior to performing microarray experiments, the stocks were backcrossed to the *w^1118^* lab stock. Unexpectedly, PCRs performed to detect presence and absence of retrotransposons proximal to three dCAP-D3-regulated gene clusters demonstrated that retrotransposon sequence was missing in dCAP-D3 mutants, but was present in *w^1118^* flies (Figure 1). In the cases of the *G2-1077* and *X-978* retrotransposons, PCR bands indicating both presence and absence were seen, suggesting that 1) the retrotransposon sequence was lost in only some of the cells and/or 2) the loss had occurred on only one of the homologous chromosomes. The *X-978*-containing locus also contained approximately 100 bp of an incomplete *INE-1* DNA transposon sequence which exhibited no loss in dCAP-D3 mutants. All of the PCRs mentioned above were also performed on wild type and dCAP-D3 mutant larvae with identical results (data not shown).

Adult flies heterozygous for a hypomorphic dCAP-D3 allele that still expresses about 10% of the levels of wild type protein [8] contained more *mdg1-1403* “presence” PCR product than adults homozygous for the hypomorphic allele (Figure 1B). Likewise, homozygous adults exhibited more “presence” product than transheterozygotes expressing the hypomorphic allele and an allele harboring a deletion of the entire dCAP-D3 locus. This data indicates that the events which cause loss of *mdg1-1403* sequence increase as dCAP-D3 expression levels decrease within the organism. Local retrotransposon loss was also seen in adult flies mutant for another Condensin II subunit, dCAP-H2 (Figure 1C), suggesting that the entire Condensin II complex, and not just dCAP-D3, acts to repress transposon mobilization *in vivo*.

The novel role described above for dCAP-D3 does not seem to be shared by members of the Condensin I complex, as flies mutant for Condensin I subunit, dCAP-D2, and SG4 cells treated with dCAP-D2 dsRNAs did not exhibit loss of retrotransposon sequence at the mdg1-1403 or G2-1077 loci. (Figure 1D and Figure S1). Finally, to confirm that acute loss of dCAP-D3 expression results in local retrotransposon loss, *Drosophila* SG4 cells were treated with dCAP-D3 dsRNA for 6 days with maximum efficiency of dCAP-D3 knock down occurring on day 4 (Figure S2A) and DNA was collected on day 6 to test for presence and absence of *mdg1-1403* (Figure 1E). Results showed that local retrotransposon loss occurred in dCAP-D3 dsRNA treated cells but not in control dsRNA treated cells (Figure 1E and Figure S2B). It should be noted that these experiments were performed in tissue culture cells due to the fact that the majority of tissue specific GAL4 drivers (and all ubiquitously expressing GAL4 drivers) cause lethality when expressed in combination with dCAP-D3 dsRNA *in vivo* (unpublished data). To confirm that complete loss of retrotransposon sequence was occurring in dCAP-D3 deficient cells/tissues, PCR amplification products corresponding to the absence product were cloned and sequenced for the *mdg1-1403, X-978, and G2-1077* retrotransposons (Figure S2C and data not shown). Sequence analyses revealed that in the majority of experiments, the entire retrotransposon had mobilized (66% of tissue culture experiments and 80% of *in vivo* experiments-not shown). Occasionally (33% of tissue culture experiments and 20% of *in vivo* experiments-not shown), a solo LTR could also be detected in the case of the *mdg1-1403* retrotransposon (data not shown). Additionally, in almost every case, a single copy of a short repeat that was normally positioned both upstream and downstream of the retrotransposon sequence remained at the locus. qRT-PCR results demonstrated that while the largest depletion of dCAP-D3 expression occurred on day 4 (Figure S2A and data not shown), precise loss of *mdg1-1403* sequence was not detectable until day 5 and dCAP-D3 levels were back to normal by day 6 (Figure S2B). The local loss of retrotransposon sequence has been reported to occur in many different organisms as a result of unequal crossing over during recombination of homologs or due to repair of a double strand break by single strand annealing [30]–[32].

### dCAP-D3 binds to the region between retrotransposon and neighboring DNA

**Figure 2:**
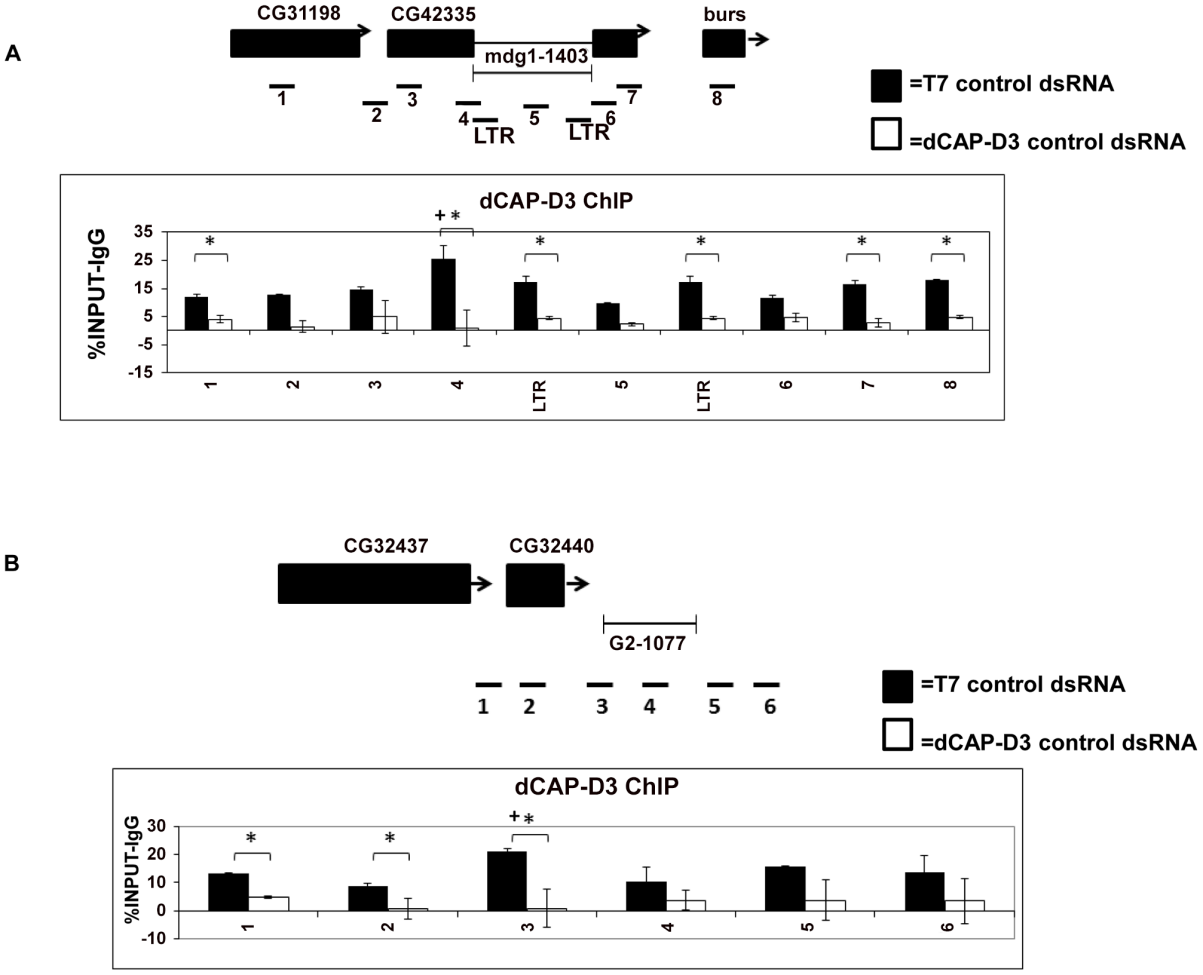
dCAP-D3 binds to loci containing retrotransposons. ChIP performed for dCAP-D3 at the (A) *mdg1-1403* locus or (B) *G2-1077* locus demonstrate binding over the entire region with the peak of binding occurring at the region encompassing both retrotransposon sequence and neighboring DNA sequence. Black bars indicate ChIP signal from SG4 cells treated with control dsRNA for 4 days and white bars indicate ChIP signal from cells treated with dCAP-D3 dsRNA for 4 days. Primer sets used are depicted above the charts. Primer sets “LTR” and “5” (*mdg1-1403* locus) and “4” (*G2-1077* locus) are not specific for each of the loci but instead prime global retrotransposon sequence. Results are the averages of 2 experiments involving duplicate IPs and are presented as a percentage of the IP with control IgG ChIP signal subtracted. (*) indicates a quantitative comparison between dCAP-D3 signal in control dsRNA and dCAP-D3 dsRNA treated cells with a p-value less than 0.05 as calculated by student unpaired t-test. (+) indicates a quantitative comparison of specific dCAP-D3 signal to the average over the entire locus with a p-value less than 0.05 as calculated by student unpaired t-test.

To understand whether dCAP-D3's ability to restrict the local loss of retrotransposon sequence was direct, Chromatin immunoprecipitation (ChIP) experiments were performed. ChIP for dCAP-D3 in cells treated with control dsRNA for 4 days demonstrated that dCAP-D3 does in fact bind to the *mdg1-1403* locus. In the Figure, (*) indicates a quantitative comparison between the indicated ChIP signal in control dsRNA and dCAP-D3 dsRNA treated cells with a p-value less than 0.05 as calculated by a student unpaired t-test. In other words, (*) indicates that the dependency of the ChIP signal on the presence of dCAP-D3 is statistically significant. (+) indicates a quantitative comparison of specific ChIP signal to the average over the entire locus with a p-value less than 0.05 as calculated by student unpaired t-test. In other words, (+) indicates that the position of the ChIP signal relative to the rest of the locus is statistically significant. In Figure 2A, the peak of dCAP-D3 binding occurred at the junction of the retrotransposon and the *CG42335* exon. Much of this binding was lost in cells treated with dCAP-D3 dsRNAs for 4 days (i.e. before loss of retrotransposon sequence, Figure S2B). dCAP-D3 binding was also seen within the retrotransposon sequence, but these results are harder to interpret, since the primers used detect all *mdg1* sequences throughout the entire genome. Interestingly, ChIP for dCAP-D3 at the *G2-1077* locus exhibited very similar results, with the peak of dCAP-D3 binding again occurring at the junction of the retrotransposon and flanking gene sequence (Figure 2B). These results suggest that dCAP-D3 does associate with different retrotransposon-containing loci and at similar places within the loci.

### dCAP-D3 regulates global levels of retrotransposon transcription in somatic cells

**Figure 3:**
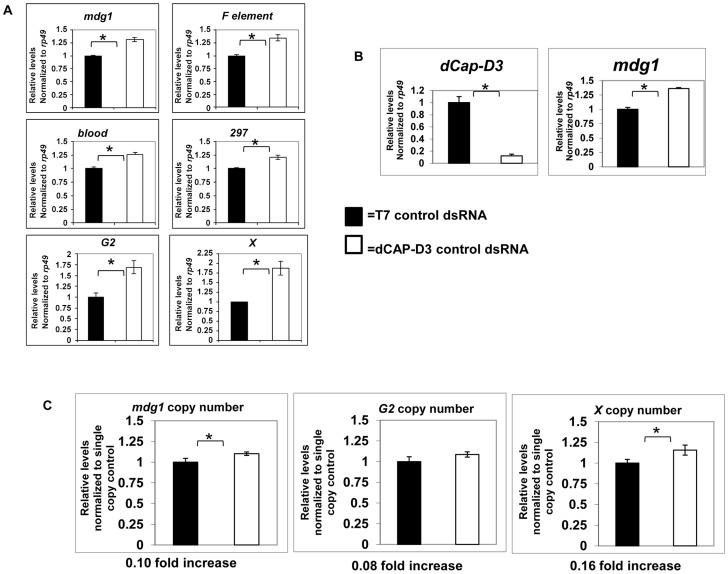
Global retrotransposon transcript levels and copy numbers increase as a result of decreased dCAP-D3 expression in somatic cells. A) qRT-PCR for 6 different retrotransposon transcripts in SG4 cells demonstrates that after 4 days of dCAP-D3 dsRNA treatment (white bars), there is a slight increase in global transcript levels as compared to cells treated with control dsRNA (black bars). B) qRT-PCR performed on cDNA from 20 wild type (*w^1118^*-black bar) or 20 *dCap-D3* mutant (*dCap-D3^c07081^dCap-D3^/Δ25^*-white bar) larval brains indicates that a significant decrease in dCAP-D3 transcripts *in vivo* results in a slight increase in *mdg1* transcripts. All qRT-PCR results were normalized to housekeeping gene *rp49* and experiments are the average of three biological replicates. C) qPCR to determine relative global copy numbers of mdg1, G2, and X element in wild type and *dCap-D3* mutant larvae indicates that copy numbers increase slightly in the mutants. Copy numbers for each retrotransposon were normalized to single copy regions located upstream. Analyses were performed on pooled samples of DNA from 10 larvae for each genotype. (*) indicates p-value less than 0.05 as calculated by student unpaired t-test.

Mobilization of retrotransposons can occur when the mechanisms that suppress their transcription fail. The transcription of an entire transposon family is inhibited at a genome-wide level through the binding of two types of small RNAs, piRNAs and endosiRNAs [33]–[37]. These small RNAs recruit proteins that help to generate a heterochromatic environment [38]–[41]. To determine whether the mechanism by which dCAP-D3 prevents loss of retrotransposon sequence involves inhibition of global retrotransposon transcription, qRT-PCR was performed for transcript levels of six different retrotransposon families. Experiments performed in SG4 cells treated with dCAP-D3 dsRNAs for 4 days showed that decreased dCAP-D3 expression resulted in small increases (1.2–1.8 fold) in global transcript levels of retrotransposons, as compared to cells treated with control dsRNAs (Figure 3A). These small changes are similar to the increases in retrotransposon transcript levels seen in SG4 cells treated with dsRNAs targeting DICER2, an enzyme shown to be necessary for generation of transposon-targeting endogenous siRNAs in *Drosophila* somatic cells [35], [42]–[45] (Figure S3). Interestingly, even though DICER2 knockdown does result in significant increases in retrotransposon transcription, it does not cause a local loss of retrotransposon sequence in these cells, suggesting that increased transcription is not sufficient by itself to observe loss of sequence from these loci. Similar results for the *mdg1* family of retrotransposons were seen *in vivo* in *dCap-D3* mutant larval brains (Figure 3B). qPCR was performed to compare copy numbers of the *mdg1, G2 and X* retrotransposons between wild type and dCAP-D3 mutant larvae. Results demonstrated small but significant increases in copy number between the two genotypes (Figure 3C). Similar increases in copy number were not observed for two single copy number genes located just upstream of the *mdg1-1403* or *G2-1077* retrotransposons (Figure S4). The small increases in retrotransposon copy numbers in *dCap-D3* mutants suggest that 1) the local loss of retrotransposon sequence is compensated for and 2) a small number of new retrotransposon copies are generated in *dCap-D3* mutants. Taken together, these results indicate that retrotransposition events may be increasing in *dCap-D3* mutants.

### Acute knockdown of dCAP-D3 results in increased DNA double strand breaks within retrotransposon sequence

**Figure 4:**
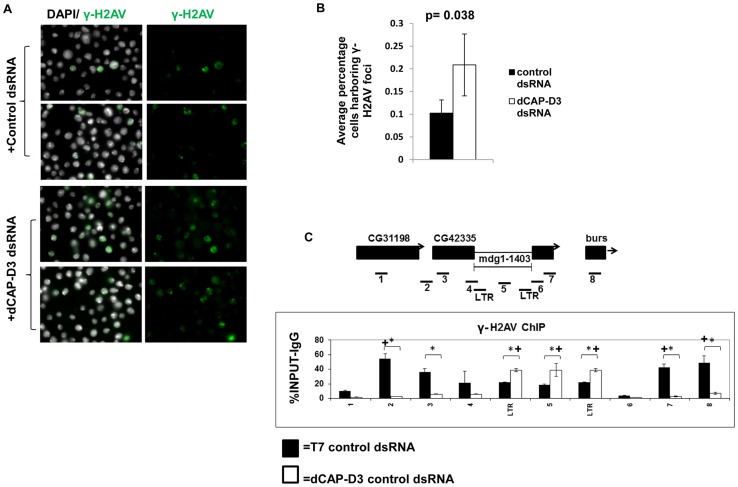
Decreased dCAP-D3 expression results in double strand break accumulation within retrotransposon sequence. A) Immunofluorescence analysis shows increased numbers of SG4 cells exhibiting γ-H2AV foci following 4 days of treatment with dCAP-D3 dsRNAs compared to cells treated with T7 control dsRNA. γ-H2AV is shown in green and DAPI stained nuclei in white. Two representative panels are shown for each dsRNA treatment. The average percentage of cells in each of 10 random frames (n≥1000 cells) harboring γ-H2AV foci is quantified in (B). C) ChIP for γ-H2AV performed on the *mdg1-1403* locus in SG4 cells treated with control dsRNA (black bars) demonstrates higher levels of binding in the regions flanking retrotransposon sequence. ChIP in cells treated with dCAP-D3 dsRNA (white bars) show a shift in γ-H2AV distribution out of retrotransposon flanking regions and into retrotransposon sequence. Primer sets used are depicted above the charts. Primer sets “LTR” and “5” are not specific for each of the loci but instead prime global retrotransposon sequence. Results are the averages of 2 experiments involving duplicate IPs and are presented as a percentage of the IP with control IgG ChIP signal subtracted. (*) indicates a quantitative comparison between γ-H2AV signal in control dsRNA and dCAP-D3 dsRNA treated cells with a p-value less than 0.05 as calculated by student unpaired t-test. (+) indicates a quantitative comparison of specific γ-H2AV signal to the average over the entire locus with a p-value less than 0.05 as calculated by student unpaired t-test.

Aside from retrotransposition, another mechanism by which retrotransposons mobilize is through homologous recombination with identical copies at allelic or non-allelic positions. The sequencing products shown in Figure S2 resemble products of single strand annealing events and/or unequal crossover between repeated sequences on the same chromosome or on homologous chromosomes, respectively [24], [46], [47]. Homologous recombination requires DNA double strand break formation for homologous sequences to recombine. γ-H2AV is a marker of DNA double strand breaks in *Drosophila* [48]. In order to determine if knock down of dCAP-D3 caused more cells to exhibit double strand breaks, we performed immunofluorescence analysis for γ-H2AV on SG4 cells treated with control or dCAP-D3 dsRNA for 4 days. Indeed, a significant increase in the percentage of cells exhibiting γ-H2AV foci was seen for cells treated with dCAP-D3 dsRNAs in comparison to cells treated with control dsRNAs (Figure 4A and 4B). Increases in double strand breaks can occur following stalling of replication forks and slowing of S phase. To determine whether acute knockdown of dCAP-D3 resulted in a change in the cell cycle distribution, FACS analysis of SG4 cells treated with control or dCAP-D3 dsRNAs was performed. Results showed that there were no dramatic changes (nothing more than 1.5% change) in cells treated with dCAP-D3 dsRNAs in comparison to control knockdown cells (Figure S5). ChIP for γ-H2AV indicated that in control dsRNA treated cells, double strand breaks at the *mdg1-1403* locus occur more frequently outside the retrotransposon sequence (Figure 4C). Surprisingly, 4 days of dCAP-D3 dsRNA treatment (before local loss of retrotransposon sequence- Figure S2B) results in a shift in the distribution of γ-H2AV, causing fewer breaks to occur outside of the retrotransposon sequence and more to occur within. Results were similar for the γ-H2AV distribution at the locus containing the *G2-1077* retrotransposon (Figure S6). These findings are surprising since the maximum level of dCAP-D3 knock-down achieved in multiple experiments was approximately 53%. This implies that even minimal decreases in dCAP-D3 levels result in major changes to the chromatin at these loci. Taken together, these data suggest that dCAP-D3 is involved in inhibiting DNA double strand break formation, especially within retrotransposon sequence.

### dCAP-D3 knockdown results in changes to chromatin structure at retrotransposon containing loci

**Figure 5:**
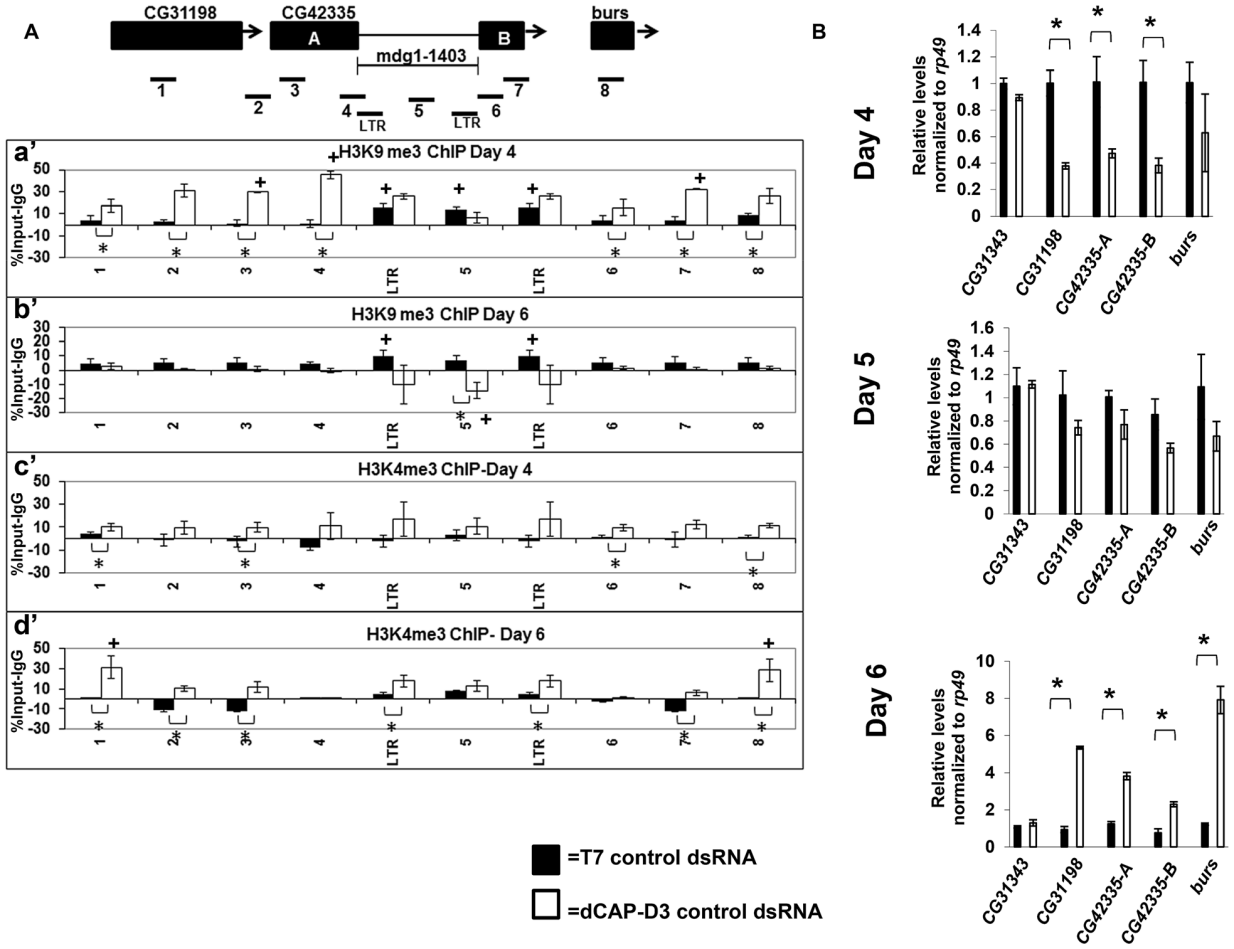
Decreased dCAP-D3 levels result in spreading of repressive histone marks and an opening of the chromatin at a dCAP-D3 regulated gene cluster containing a retrotransposon. A) ChIP for trimethylated H3K9 (a' and b') and trimethylated H3K4 (c' and d') performed on the *mdg1-1403* locus in SG4 cells treated with control dsRNA (black bars) demonstrates absent or low levels of the marks in the areas surrounding the retrotransposon but high levels of H3K9me3 within retrotransposon sequence. ChIP for trimethylated H3K9 in cells treated with dCAP-D3 dsRNA demonstrates a dCAP-D3 dependent increase of the mark in the areas surrounding the retrotransposon (a') and a dCAP-D3 dependent decrease of the mark in within retrotransposon sequence following retrotransposon mobilization (b'). ChIP for trimethylated H3K4 in cells treated with dCAP-D3 dsRNAs shows a dCAP-D3 dependent increase over the entire locus prior to mobilization (c') which persist following mobilization. Primer sets used are depicted above the charts. Primer sets “LTR” and “5” are not specific for each of the loci but instead prime global *mdg1* sequence. Results are the averages of 2 experiments involving duplicate IPs and are presented as a percentage of the IP with control IgG ChIP signal subtracted. (*) indicates a quantitative comparison between indicated ChIP signal in control dsRNA and dCAP-D3 dsRNA treated cells with a p-value less than 0.05 as calculated by student unpaired t-test. (+) indicates a quantitative comparison of specific ChIP signal to the average over the entire locus with a p-value less than 0.05 as calculated by student unpaired t-test. (B) qRT-PCR for transcript levels of genes surrounding *mdg1-1403* (as depicted in diagram in A) indicates that compared to control dsRNA treated cells, transcription is decreased in dCAP-D3 dsRNA treated cells prior to retrotransposon mobilization (day 4) and increases to almost normal levels on the day of retrotransposon mobilization (day 5). Following retrotransposon mobilization (day 6), transcription increases more than 2-fold in dCAP-D3 dsRNA treated cells. CG31343 is positioned approximately 12 kb upstream of *mdg1-1403*. Results are the average of three experiments and (*) indicates p-value less than 0.05 as calculated by student unpaired t-test.

Increased levels of double strand breaks have been shown to lead to an opening of the chromatin structure in order to facilitate repair [49]–[51]. To examine whether the increased levels of double strand breaks within retrotransposon sequence in dCAP-D3 dsRNA treated cells also resulted in an opening of the chromatin structure, ChIP assays to detect histone modifications were performed. In order to better understand the timing of changes in histone marks in reference to the precise loss of retrotransposon sequence, these assays were done in the context of the SG4 time course experiments presented in Figure S2. ChIP was performed on the *mdg1-1403* locus to examine levels of the repressive trimethylated H3K9 mark (Figure 5A, a' and b') and the activating trimethylated H3K4 mark (Figure 5A, c' and d'). Results of experiments performed in SG4 cells treated with control dsRNAs revealed significant levels of H3K9 trimethylation only within the retrotransposon sequence (Figure 5A, a' and b', black bars). High levels of H3K9 trimethylation have been reported previously at *Drosophila* retrotransposon sequences [40], [52]. No significant levels of H3K4 trimethylation were detected at the locus in cells treated with control dsRNAs (Figure 5A, c' and d', black bars). dCAP-D3 knockdown at a time point prior to local loss of sequence, significantly increased levels of H3K9me3 at the sequences surrounding the *mdg1-1403* retrotransposon (Figure 5A, a' white bars). Transcription of the surrounding genes was correspondingly decreased (Figure 5B, top panel). qRT-PCR for the CG31343 gene, located approximately 12 kb upstream from the 3′ end of the *mdg1-1403* retrotransposon, was performed as a negative control and demonstrates no significant change in transcription. Following loss of retrotransposon sequence in dCAP-D3 knockdown cells, H3K9me3 marks actually decreased within the retrotransposon sequence (Figure 5A, b', white bars). Recently, ChIP-seq experiments showed that repressive H3K9me3 marks found at *mdg1* retrotransposons in *Drosophila* somatic cells are held within strict boundaries and, on average, do not extend into neighboring regions [40]. Therefore, this data suggests that decreases in dCAP-D3 expression may cause a loss of repressive boundary and a local spreading of H3K9me3 from within the retrotransposon sequence into the surrounding sequence. Also, prior to the local loss of retrotransposon sequence, significant increases in H3K4me3 levels were seen over the entire locus in dCAP-D3 dsRNA treated cells (Figure 5A, c', white bars). Transcription of the surrounding genes also increased and returned to basal levels on the day that the local loss of retrotransposon sequence occurred (Figure 5B, middle panel). The appearance of H3K4me3 marks in dCAP-D3 dsRNA treated cells prior to retrotransposon mobilization suggests that the increases in double strand breaks within *mdg1* retrotransposon sequence may indeed lead to a local opening of chromatin. Finally, ChIP results show that the increase in H3K4me3 in areas surrounding the retrotransposon persisted following retrotransposon mobilization suggesting that dCAP-D3 knockdown may in fact cause a permanent change in chromatin structure.

### dCAP-D3 prevents pairing of homologous retrotransposon-containing loci

**Figure 6:**
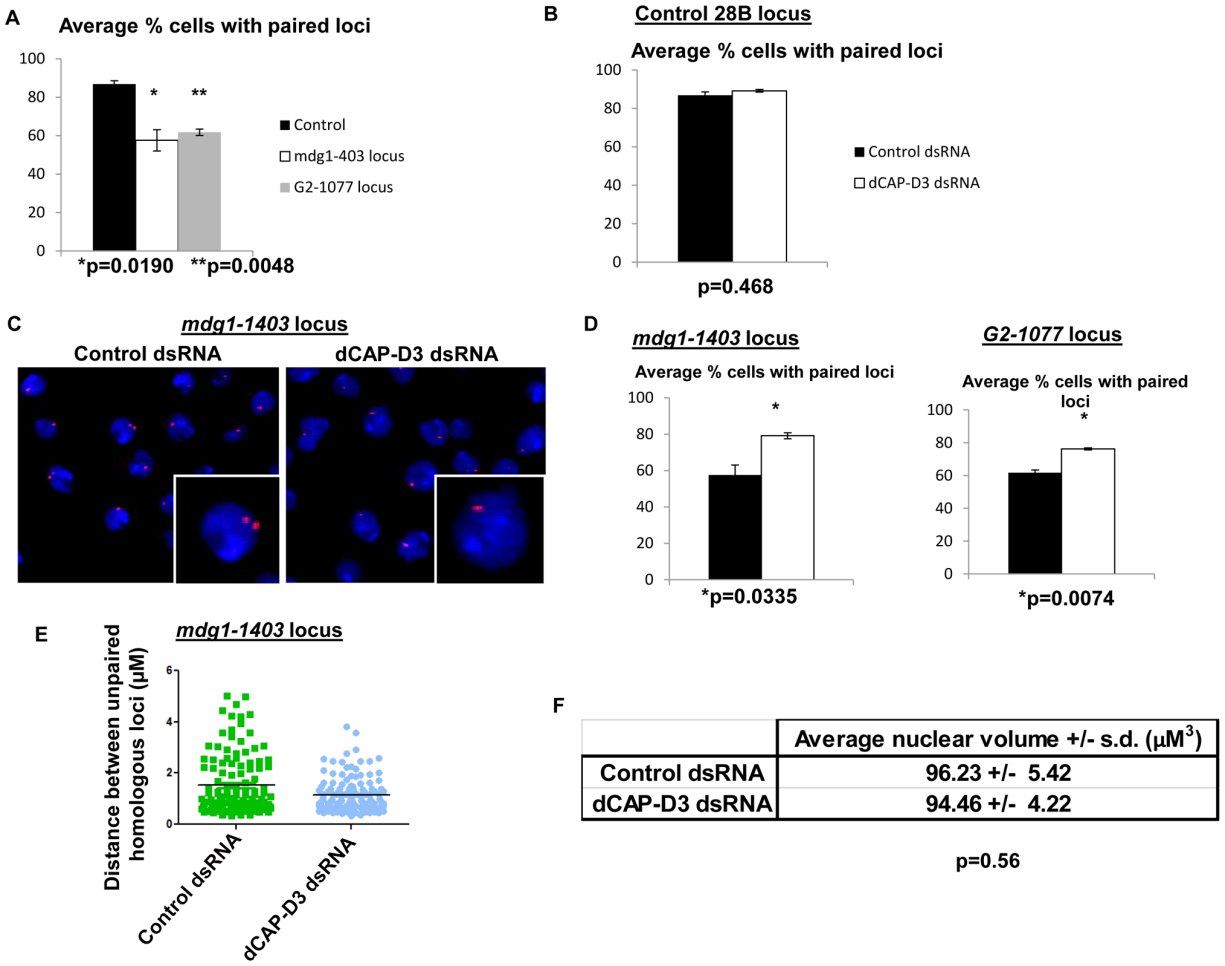
Pairing of retrotransposon loci on homologous chromosomes is increased in cells treated with dCAP-D3 dsRNAs. A) Quantification of the percentage of cells harboring single FISH dots representing paired loci in SG4 cells treated with control dsRNAs for 4 days shows that the control 28B euchromatic locus (black bar) which is not proximal to retrotransposon sequence is paired at a significantly higher frequency than the *mdg1-1403* locus (white bar) or the *G2-1077* locus (grey bar). B) Quantification of the percentage of cells harboring single FISH dots representing paired loci demonstrates a slight but not significant increase in pairing of the 28B locus in dCAP-D3 dsRNA treated SG4 cells (white bar) compared to T7 control dsRNA treated cells (black bar). C) FISH experiments using probes hybridized to the *mdg1-1403* locus demonstrate a significantly higher frequency of single FISH dots/pairing in cells treated with dCAP-D3 dsRNA for 4 days, as compared to cells treated with control dsRNA for 4 days. Probes were labeled with Alexa-555 and DAPI-stained nuclei are shown in blue. Inset boxes are magnifications of a single cell within the larger image. D) Quantification of the percentage of cells harboring single FISH dots from FISH experiments using probes hybridized to the *mdg1-1403* locus (left) and *G2-1077* locus (right) demonstrate significantly higher frequencies of pairing in cells treated with dCAP-D3 dsRNA (white bars) as compared to cells treated with control dsRNA (black bars). E) The distance between unpaired homologous *mdg1-1403* loci in control dsRNA (green, n = 124) and dCAP-D3 dsRNA (blue, n = 135) treated SG4 cells was measured by manually planing through z stacks of images taking on a confocal microscope. The mean of each sample set is shown as a black horizontal line. For each FISH experiment/quantification in (A–D), over 200 cells were counted using the maximum projections and experiments were repeated three times. Only non-mitotic cells were counted. F) Nuclear volumes of cells treated with control or dCAP-D3 dsRNAs were measured using the Volocity software and changes were found to be not statistically different between the two populations. 5 separate fields of view containing at least 50 cells (two independent experiments) were counted for each population. Only non-mitotic cells were counted.

Pairing of homologous chromosomes is a phenomenon which occurs throughout the cell cycle in *Drosophila* somatic cells and has been suggested to be the reason why these cells favor the homologous chromosome as a template for repair of double strand breaks [53]. Recently, Condensin II was characterized as an “anti-pairing” complex and loss of dCAP-H2 or dCAP-D3 was shown to increase the frequency of pairing at a number of heterochromatic loci in *Drosophila* tissue culture cells [15]. Combined with increases in double strand breaks in retrotransposon sequence, an increase in pairing of homologous chromosomes could lead to increased levels of recombination between retrotransposons. Each of the loci that exhibited retrotransposon mobilization in dCAP-D3 mutants was present in euchromatic regions of the genome. Therefore, to understand whether the ability of dCAP-D3/Condensin II to prevent homolog pairing was involved in its ability to restrict transposon mobilization, it was necessary to first determine the “normal” frequency of pairing of specific dCAP-D3 regulated retrotransposon-containing loci. FISH experiments were performed on cells treated with dCAP-D3 dsRNA for 4 days (prior to local loss of retrotransposon sequence) using three different PCR amplified probes which hybridized to: 1) a euchromatic “control” region adjacent to the 28B cytological location that was annotated to be positioned 50 kb away from any known retrotransposon sequences; 2) a 12 kb region immediately upstream of the *mdg1-1403* retrotransposon containing locus; or 3) a 10 kb region immediately upstream of the *G2-1077* retrotransposon containing locus. Repeated experiments demonstrated that while the euchromatic control regions on homologous chromosomes paired 87% of the time in SG4 cells, the *mdg1-1403* and *G2-1077* retrotransposon containing loci were paired only 57% and 62% of the time, respectively (Figure 6A). This suggests that dCAP-D3 regulated, retrotransposon-containing, euchromatic loci are normally less paired than euchromatic loci which are not proximal to retrotransposons. While 4 days of dCAP-D3 dsRNA treatment of these cells did result in a slight increase in pairing at the euchromatic 28B control region, the difference was not statistically significant (Figure 6B). However, the pairing of the two retrotransposon-containing loci increased dramatically and significantly (Figure 6C–6D). Additionally, the majority of unpaired *mdg1-1403* loci in dCAP-D3 dsRNA treated cells were found to be closer in distance to one another in comparison to unpaired loci in control dsRNA treated cells (Figure 6E). 8–13% of SG4 cells are aneuploid according to FACS analysis, independent of dCAP-D3 levels (Figure S5). To rule out the possibility that a decrease in nuclear volume could be the reason for increased homolog pairing, nuclear volume was measured in control and dCAP-D3 dsRNA treated cells using the Volocity software (Figure 6F). Results indicated no significant change in average nuclear volume between the two cell populations.

**Figure 7:**
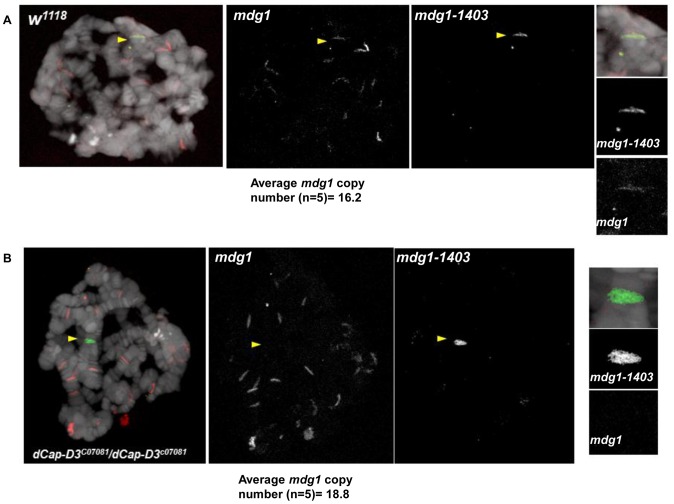
*dCap-D3* mutant salivary glands exhibit loss of *mdg1-1403* and increases in retrotransposon copy number at other loci. FISH experiments using probes hybridized to the *mdg1-1403* locus (green) and to *mdg1* retrotransposon sequence (red) demonstrate that wild type glands retain the *mdg1* retrotransposon sequence at the *mdg1-1403* locus (middle panel and smaller panels on right in A) while *dCap-D3* mutants do not (middle panel and smaller panels on right in B). Yellow arrows indicate co-localization of probes in wild type preparations and absence of co-localization in *dCap-D3* mutant preparations. The average copy number for each genotype was determined for salivary glands from 5 separate larvae by counting total numbers of *mdg1* bands. Salivary gland chromatin was stained with DAPI and is shown in white. Additional FISH experiments are shown in Figure S7.

To confirm the specificity of our FISH probes and to visualize the chromatin at dCAP-D3-regulated retrotransposon containing loci *in vivo*, FISH was also performed on salivary gland squashes from wild type and dCAP-D3 mutant larvae. *Drosophila* salivary glands contain polytene chromatin which is formed by continuous endoreduplication of chromatids which then pair together. Homologous chromosomes (estimated to each contain over 500 copies of DNA) also pair, creating the beautiful banding pattern that polytene chromosomes are famous for. In the FISH experiments presented in Figure 7, two probes were used: an Alexa 555 (red) labeled probe which hybridized to the multi-copy *mdg1* retrotransposon sequence and an Alexa 488 (green) labeled probe which hybridized to the single copy region just upstream of the *mdg1-1403* retrotransposon. In agreement with previous PCR results, the mdg1 and mdg1-1403 probes co-localized in wild type larvae, indicating presence of the *mdg1-1403* retrotransposon on both homologs (Figure 7A and Figure S7A). FISH analyses performed on dCAP-D3 mutant salivary gland squashes showed that the mdg1 and mdg1-1403 probes did not co-localize, confirming that a local loss of mdg1-1403 retrotransposon sequence had indeed occurred (Figure 7B and Figure S7B). The average *mdg1* copy number (5 larvae examined per genotype) was also determined by counting the number of bands that the mdg1 probe hybridized to. The average copy number in wild type larvae was 16.2 and in dCAP-D3 mutants was 18.8. Therefore, FISH analyses suggest a 1.16 fold increase in mdg1 copy number in dCAP-D3 mutants and this is very close to the 1.1 fold increase seen by qPCR (Figure 3). Together, the FISH results in *Drosophila* somatic tissue culture cells and tissues support the idea that dCAP-D3/Condensin II prevents pairing of homologous chromosomes and restricts the movement of retrotransposons within the genome.

## Discussion

### A possible model for how dCAP-D3/Condensin II acts to prevent retrotransposon mobilization

**Figure 8:**
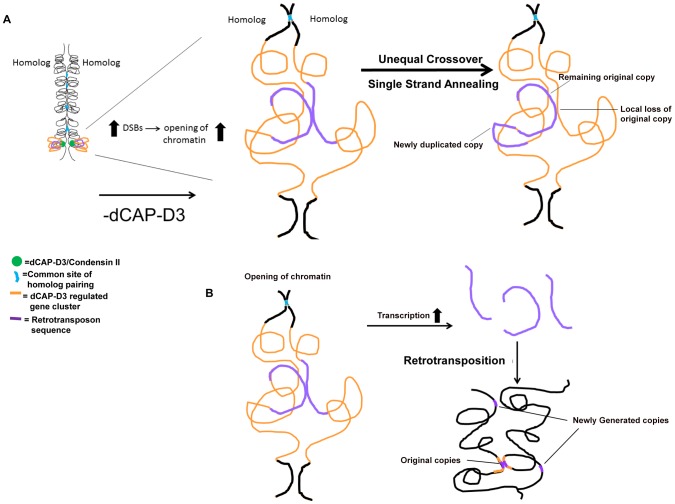
Possible model for how dCAP-D3/Condensin II might restrict retrotransposon mobilization in *Drosophila* somatic cells. (A) In this model, dCAP-D3/Condensin II (green circles) organizes gene clusters that it transcriptionally regulates (orange) into a rigid and possibly looped structure (shown on the left). This would serve to position retrotransposon sequences within the cluster (purple) in a manner that is inhibitory to recombination with the homologous chromosome. A decrease in dCAP-D3 levels would result in increased DNA double strand breaks (DSBs) within retrotransposon sequence and an opening of the chromatin structure. This would then allow retrotransposon sequences to more frequently contact the homologous chromosome (magnified image of dCAP-D3 regulated gene cluster shown on the right). While having only minor effects on regions that normally pair at high frequencies (blue), the increased frequency of contacts between repeats flanking retrotransposon sequences on homologous chromosomes combined with increased double strand breaks could lead to unequal crossover events and/or repair by single strand annealing. This would then result in loss of locus-specific retrotransposon sequence and gain of sequence in a separate place. (B) It is also possible that, at some retrotransposon containing dCAP-D3 regulated gene clusters, the opening of chromatin and slight increase in retrotransposon transcription seen following decreased dCAP-D3 expression could lead to generation of RNA intermediates and retrotransposition of new copies to other places in the genome.

In this manuscript we show that decreased levels of dCAP-D3/Condensin II lead to retrotransposon mobilization within specific gene clusters shown to be transcriptionally regulated by dCAP-D3. In tissue culture cells, our results demonstrate that homologous retrotransposon containing clusters remain largely unpaired which is in striking contrast to homologous euchromatic loci that do not contain retrotransposon sequences. Interestingly, the mobilization events detected both *in vivo* and *in vitro* resulted in either the retention of a single LTR at the locus or a precise loss of retrotransposon sequence in one locus and a small increase in copy number elsewhere in the genome. In the model presented in Figure 8, we put forth the hypothesis that dCAP-D3/Condensin II mediated looping of chromatin at homologous, euchromatic, retrotransposon containing loci holds the regions at distances great enough to prevent recombination. In dCAP-D3 deficient cells, this rigid chromatin structure is not maintained, possibly leading to increased double strand breaks within retrotransposon sequence. This in turn would cause an opening of chromatin in the region and would give homologous retrotransposon containing loci more of an opportunity to pair (Figure 8A). Repair mechanisms that would lead to a local loss of retrotransposon sequence at one of the loci and a gain of a copy elsewhere in the genome include repair by the single strand annealing pathway or unequal crossover events between the small repeats found before and after the retrotransposon sequence. While these types of recombination repair do explain the local loss of sequence, they do not explain the small increase in copy number seen in dCAP-D3 deficient cells. Therefore, we also propose that, as a result of the opening of the chromatin at these loci, transcription increases and allows retrotransposon encoded retrotransposase enzyme to be made and generate additional copies (Figure 8B). These new retrotransposition events would allow both original copies to remain in their loci and new copies to be generated and insert elsewhere. Supporting evidence for a role of Condensin II in regulating homologous crossover events comes from a recent study in *C. elegans* that worms heterozygous for Condensin II subunits exhibited increases in double strand breaks, increases in crossover events, and increases in X chromosome axis length in meiotic tissue [54]. The differential placement and number of double strand breaks in the *C. elegans* Condensin mutants were hypothesized to be caused by the changes in axial chromatin structure since axis lengths did not change in response to varying numbers of double strand breaks between mutants. Loss of *Drosophila* Condensin II subunits also lead to axial expansion [14], [55], [56]. Interestingly, the *mdg1-1403* locus appears expanded in the dCAP-D3 mutants (Figure 7, Figure S7), and it is possible that this local expansion and change in chromatin structure could be the cause of the repositioning of double stand breaks shown in Figure 4. Finally, while we do not discuss it in our model, the loss of Condensin II expression results in disorganization of chromosome territories and intermingling of chromosomes in *Drosophila* cells [55]. Therefore, it is also possible that the frequency of recombination between retrotransposon sequences on different chromosomes could increase, leading to loss of the remaining retrotransposon copy on one of the homologs in cells deficient for dCAP-D3.

It should be noted that recently published IP/mass spectrometry data from both ovary extracts and embryo extracts could not identify physical interactions between SMC2 and dCAP-D3 or dCAP-H2, calling into question the existence of a *Drosophila* Condensin II complex [57]. However, the authors of this study do acknowledge the possibility that the *Drosophila* Condensin II complex may only form on chromatin, and therefore may not be picked up by their assays. Given that 1) dCAP-D3 and dCAP-H2 have been shown to be physical members of Condensin II in other organisms, 2) that the phenotypes which result from loss of expression of these subunits in *Drosophila* are almost identical and 3) that dCAP-H2 overexpression phenotypes have been shown to be dependent on dCAP-D3 [16], [56], we will continue to label them as such until an extensive analysis of dCAP-D3 interaction partners involving multiple tissues, retention of chromatin dependent interactions, and testing of specific *dCap-D3* mutants has been performed.

### Relationship between Condensin II transcriptional regulation and prevention of retrotransposon mobilization

The minor, but significant increases in retrotransposon transcript levels in somatic tissues and cells expressing lower levels of dCAP-D3 suggest that dCAP-D3 regulates global retrotransposon transcript levels. We have previously shown that dCAP-D3 regulates transcription of many genes in *Drosophila* larvae and adults, but the mechanism remains unclear [8]. The experiments in SG4 cells show that dCAP-D3 binds close to the junction between retrotransposon and neighboring DNA sequence. They also demonstrate that dCAP-D3 is necessary for maintaining basal transcription levels of retrotransposon-containing gene clusters prior to local loss of retrotransposon sequence. If dCAP-D3 acts to set up boundaries between a retrotransposon and neighboring DNA sequence, then binding sites located within the neighboring sequence could confer local specificity. In support of this, our data show an increased spreading of repressive H3K9me3 marks into the area surrounding *mdg1-1403* in dCAP-D3 dsRNA treated cells. This data is also consistent with earlier findings that dCAP-D3 is a suppressor of Position Effect Variegation in somatic tissues [7]. Alternatively, the temporary increase in H3K9me3 at the locus prior to loss of retrotransposon sequence could be due to the increase in homolog pairing in dCAP-D3 knock down cells; silencing of extrachromosomal copies of genes proximal to transposons has been shown to increase when these regions pair [58]. Transcription of genes surrounding *mdg1-1403* increases above basal levels in dCAP-D3 dsRNA treated cells once the retrotransposon sequence is lost. Interestingly, even when dCAP-D3 expression levels return to normal, the increased transcription and increased levels of active H3K4me3 marks at the locus remain. It is also interesting to note that the band recognized by the mdg1-1403 probe in the dCAP-D3 mutant polytene chromatin squashes appeared longitudinally thicker and less condensed (Figure 7B and Figure S7B). This supports our model and suggests that the presence of the retrotransposon within the locus elicits a dCAP-D3-dependent structural configuration that is lost when the retrotransposon sequence is lost.

### Links between dCAP-D3/Condensin II and repair of double strand breaks

Results presented here show that dCAP-D3 prevents increased γH2AX localization in retrotransposon sequence. Interestingly, human Brd4 isoform B was recently reported to bind to SMC2 and CAP-D3 proteins, and SMC2 was shown to be necessary for Brd4's ability to maintain a more condensed chromatin structure and inhibit DNA damage signaling following gamma irradiation [59]. This suggests 1) that the functions of Condensin II in DNA damage repair may be conserved in human cells, and 2) that Condensin II's role in repair most likely requires its ability to maintain rigid chromosome structure and organization. Recently, a role for Condensins in organizing retrotransposons within the nucleus was reported in yeast. Retrotransposons cluster in yeast and it was demonstrated that the Non-Homologous End Joining (NHEJ) repair associated Ku proteins as well as Condensin were both necessary for the observed clustering [60]. The reported association between DNA repair proteins and Condensin is intriguing and might suggest, if the interaction was conserved in flies, that Condensins play a role in the actual repair of double strand breaks at retrotransposon sequences. However, we do not see mass clustering of the *mdg1-1403* retrotransposon in *Drosophila* cells and the studies presented here show that in *Drosophila*, Condensin-associated mechanisms exist to prevent retrotransposons on homologous chromosomes from coming into close contact. Furthermore, our sequencing results indicate that either single strand annealing or unequal crossover events have occurred in dCAP-D3 mutants, instead of NHEJ mediated repair. These discrepancies might be attributed to the high degree of homologous chromosome pairing throughout the cell cycle in *Drosophila*. In fact, single strand annealing (even over NHEJ) has been shown to be the dominant double strand break repair pathway at transposon containing loci in *Drosophila* when direct repeats flank a double strand break [31]. Additionally, yeast only possess Condensin I and not Condensin II, so it is possible that Condensin II has diverged to have different functions or even to antagonize Condensin I function at retrotransposon sequences.

Interestingly, ChIP for phosphorylated H2AX in human cells expressing SMC2 RNAi showed that double strand breaks occur frequently within LTR sequences and a type of non-LTR retrotransposon, SINES [61]. Therefore, the ability of Condensin II to prevent double strand break accumulation and recombination within retrotransposon sequence may not be unique to *Drosophila* Condensin II. This has important implications for Condensin II as a possible tumor suppressor in human cells. Various types of tumor cells have been found to harbor mutations in Condensin II proteins including CAP-D3 (COSMIC database- http://cancer.sanger.ac.uk/cancergenome/projects/cosmic/). While somatic homolog pairing is not as prevalent in human cells as in *Drosophila*, certain instances of abnormal pairing have been implicated in the generation of tumors [62], [63]. Further studies will be necessary to elucidate whether uncontrolled retrotransposon recombination and/or retrotransposition might play a role in the generation of genomic instability in human cells deficient for or expressing mutant Condensin II proteins.

## Materials and Methods

### Fly strains

*w^1118^* flies were used as “wild type” controls for microarray experiments. Unless otherwise noted, the genotype of *dCap-D3* mutants was a transheterozygous combination of *dCap-D3^Δ25^/dCap-D3^c07081^* which was obtained by mating *dCap-D3^Δ25^/CyO, GFP* virgins to *dCap-D3^c07081^/CyO, GFP* males at 25°C. *Cap-H2* mutant stocks (*Cap-H2^Z3-0019^* and *Cap-H2^Z3-5163^*) were a generous gift from Dr. Giovanni Bosco. The *dCap-D2* mutant stock (*dCap-D2^f03381^*) was obtained from the Exelixis Collection. All flies were maintained at 25°C and placed in vials containing standard dextrose medium.

### Cell culture & dsRNA cell treatment

SG4 cells, obtained from the *Drosophila* Genomics Research Center, were grown in Shields and Sang M3 Insect Medium supplemented with 10% fetal bovine serum (FBS) and 1% penicillin/streptomycin. Primers used for dsRNA sequence amplification were:

dCAP-D3 dsRNA = Forward primer: CTAATACGACTCACTATAGGGAGTGCAGATTACGTGCTGGAAGC, Reverse primer: CAGGGGATTGACTAGGACCAG

dCAP-D2 dsRNA = Forward primer: CTAATACGACTCACTATAGGGAGCTTCCAGATCTTGGGCACAT, Reverse primer: CGAGCTCTTGTCTTCCAACCT7

DICER2 dsRNA = Forward primer: CTAATACGACTCACTATAGGGAGCTGCCCATTTGCTCGACATCCCTCC, Reverse primer: TTACAGAGGTCAAATCCAAGCTTG

control primers were from the Ribomax Large Scale RNA Production System (Promega). 100 µL of PCR product was purified using the PCR Purification Kit (Qiagen). The Ribomax Large Scale RNA Production System was used, according to manufacturer's protocol, to produce control T7 and dCAP-D3 dsRNA for cell treatment. SG4 cells were plated in 6-well dishes at 1×10^6^ cells/mL and kept at RT for 1–2 h. Cells were then soaked in 1 mL Express Five SFM (Invitrogen) with 1% FBS containing 50 µg dsRNA for 2 hours at RT. 2 mL M3 Media with serum was then added to the well. The procedure was repeated 48 hours later. Plates were covered in parafilm, kept at 25°C and collected at indicated time points.

### qRT–PCR analyses

Experiments were performed as described in [8]. Briefly, TRIzol (Invitrogen) was used to harvest total RNA from tissues and cells according to manufacturer's protocol. After RNA was purified using the RNAeasy kit (Qiagen), the Taqman Reverse Transcription kit (Applied Biosystems) was used to reverse transcribe 1.5 µg of RNA into cDNA. qRT-PCR was performed using the Roche Lightcycler 480 to amplify 15 µL reactions containing .5 µL of cDNA, .5 µL of a 10 µM primer mix and 7.5 µL of SYBR Green Master Mix (Roche). For qRT-PCR experiments involving larval tissues, three groups of 10 larvae per genotype were used. For experiments involving SG4 cells, three groups of 1.5×10^6^ SG4 cells treated with T7 dsRNA and dCAP-D3 dsRNA were used. Three independent experiments were performed in all cases.

Primer sequences used were:

dCAP-D3 F1 CGTGCTGTTGCTTTACTTCGGCC

dCAP-D3 R1 GGCGCATGATGAAGAGCATATCCTG

CG31343 F1 CACCTTCTCGTACGCCAAGCC

CG31343 R1 CCTGGAAGGACGCAAATAGATCCC

CG31198 F1 CAGTAACACCCGTCTGATCTCATCG

CG31198 R1 CGGGCCACGAACTCGGAAATTATG

CG42335A F1 CAGATTGTTCGGCCCAACGGG

CG42335A R1 CATGAAGGCCAGCAGATATGTGGAC

CG42335B F1 GGAGAATCCTGACTTGGTTCAGGC

CG42335B R1 GTGGTGAAGTACTCCGCCATGTC

Mdg1 F1 AACAGAAACGCCAGCAACAGC

Mdg1 R1 TTTCTGATCTTGGCAGTGGA

Blood F1 CCAACAAAGAGGCAAGACCG

Blood R1 TCGAGCTGCTTACGCATACTGTC

297 F1 GGTGATCCAGAAACCCTTCA

297 R1 CTTTCGATGGCTCCCAGTAG

F-element F1 TCATCTTCCATCGTTGTGGA

F-element R1 CACATTCTGCAGTTCGCTTC

G2 –element F1 GAGCTCGAGATTCCATGGGTAGAC

G2-element R1 GCGTTCTCTGCAGGCGTCTTAG

X-element F1 GCCAGCCTGCAACAGGTTGAAG

X-element R1 CTCTGGCGCACAATGACTTCGG

### Genomic PCR

DNA from whole fly, larval tissues, and SG4 cells was extracted and purified using DNAzol (Invitrogen). 5 whole adult flies, 10 larval salivary glands, and 1.5×10^6^ SG4 cells were suspended in 1 mL DNAzol. Flies and salivary glands were homogenized using a pestle grinder and SG4 cells were vortexed. Tubes were centrifuged at 10000 g for 10 min at 4°C and the supernatant was transferred to a new tube. DNA was precipitated by the addition of 500 µL of 100% ethanol. The reaction was kept at RT for 1–3 min and then centrifuged at 10000 g for 5 min at 4°C. The supernatant was discarded and the remaining DNA pellet was washed twice with 700 µL 70% ethanol. After drying, DNA was resuspended in 50–100 µL H2O. PCR was performed with the extracted DNA, primers listed below and GoTaq (Promega). Equal DNA concentrations were used for control and experimental samples. PCR reactions were run using the Mastercycler pro (Eppendorf). Final PCR products were observed on a gel and imaged using the ChemiDocTM XRS+ Imager (Bio-Rad). Control primers used were:

Tubulin forward: CGCGCGGTGCTCTTGGACTTGGAACCG, Tubulin reverse: GCTTGTCATACTGGTTGAGAGCTCGCTCG. To detect presence of mdg1-1403: forward GAATACCGGTTGAGAACCGTGC, reverse GGACCACCCTAATTCCTTAGGGTC. To detect absence of mdg1-1403: forward GAATACCGGTTGAGAACCGTGC, reverse CCGGCGATGGTACTTCATGACC. To detect presence of the G2-1077: forward GTGATTAATGGGCGCGTCATTG, reverse CTGCTGTAAACAGGGTGTAGAGG. To detect absence of the G2-1077: forward GTGATTAATGGGCGCGTCATTG, reverse CTTGCCTCTAAGGTTATCCTAAGC. To detect presence of the X-978: forward GTCGCTATCCAACAAGCTG, reverse CTATTGAATCGCTTTGTTC

To detect absence of the X-978: forward GCTTGCATTCAAGAGATACC, reverse CTATTGAATCGCTTTGTTC.

### Cloning & sequencing of PCR products

PCR products were purified using the Qiaquick Gel Extraction Kit (Qiagen) following manufacturer's protocol. 4 µL purified DNA was cloned into a pCR4-TOPO TA Vector using the TOPO TA Cloning Kit (Life Technologies), following manufacturer's protocol.

After transformation of DNA into the TOPO vector, cells were plated on agar plates with carbenicillin (at 50 µg/mL) and grown overnight at 37°C. Colonies were selected and incubated in 5 mL LB Broth with 50 µg/mL carbenicillin for 12–14 hours at 37°C with constant shaking. The incubated colonies were then purified using the Wizard Plus Minipreps DNA Purification System (Promega). PCR was performed to screen for positive clones. Verified products were sent for sequencing to the Genomics Core of the Cleveland Clinic Lerner Research Institute. Primers used to amplify the cloned sequence were M13 forward; GTAAAACGACGGCCAG, and M13 reverse; CAGGAAACAGCTATGAC.

### qPCR

Total genomic DNA was isolated from 20 larvae for each genotype. 50 ng of DNA was used per reaction and primers were present at 5 mM concentrations. PCR reactions were carried out as described in [64]. Primers used to amplify G2 copy numbers were 5′cgcctaaagcaactccactggc3′ AND 5′gcttgcagtgccacacagctg3′ and were normalized to an upstream single copy control region using primers 5′ctcggccctaaattgtccgttcg3′ AND 5′ctgctagctaatccgcgcttctc3′. Primers used to amplify mdg1 copy numbers were 5′gaccattggggtggtggagtg3′ AND 5′gcgatctgagtgagtagagtgtcag3′and were normalized to an upstream single copy control region using primers 5′gcaatggagaactggggtctgttg3′ AND 5′catgtgcgcctgttcgtgagc3′. Primers used to amplify×element copy numbers were 5′ GCCAGCCTGCAACAGGTTGAAG3′ AND 5′ CTCTGGCGCACAATGACTTCGG3′ and were normalized to an upstream single copy control region using primers 5′ CCGGATTCTTACTTGCCACGCC3′ AND 5′ CAAATTGCGCGCAAAAGAAGCCGTG3′.

### Cell cycle analysis

Cells were harvested from one well of a 6 well plate following dsRNA treatment and washed in 5 mL cold PBS. Following resuspension in 0.5 mL of cold PBS, 4.5 mL 95% ethanol was added while gently vortexing. Cells were incubated overnight at 4°C and then resuspended in 2 mL of 1× Propidium Iodide (PI)/RNAse solution (1× PI, 1× RNAse in PBS with 10% FBS) made from 50× stock solution of PI (.5 mg PI per mL of 38 mM Sodium Citrate pH 7.0 and 40× stock solution of RNAse (10 mg/mL RNAse A in 10 mM Tris-HCL pH 7.5+15 mM NaCl boiled 15 minutes and cooled at RT. Following overnight incubation in PI/RNAse solution at 4°C, cells were analyzed on a BD Biosciences FACSCalibur Flow Cytometer. Cell cycle distributions were computed using the ModFit software.

### Immunofluorescence analysis of cells and salivary glands

Primary antibodies included YZ384-dCAP-D3 (previously generated for our lab) and anti-GFP (Jackson Immunoresearch). Briefly, SG4 cells were washed with 1× PBS, fixed using a 4% paraformaldehyde solution for 10 min at RT, washed again with 1× PBS, incubated in 0.2% Triton X-100 for 5 min on ice, and washed again with 1× PBS. The cells were then treated with blocking buffer (0.5% NP-40, 1% BSA in 1× PBS) for 30 min at RT. Cells were incubated with γH2AV Antibody at 1∶500 (generous gift from Dr. Kim McKim) for 1 h at RT. Three 1× PBS washes followed, each for 5 min at RT with gentle shaking. Cells were mounted on slides using VectaShield with DAPI (Vector Labs), and sealed. To quantify the number of cells positive for γH2AV, 10 separate, random fields containing at least 100 cells in each field were counted. Imaging was performed on a Zeiss AxioImager Z1 motorized epifluorescent microscope using the ApoTome System and a MRm CCD camera.

### FISH experiments

Probes were made by PCR amplification using primers designed against 1–1.5 kb gene regions, totaling 12–15 kb of total DNA sequence. PCR reactions were run on an agarose gel and bands were extracted using the QiaQuick Gel Extraction Kit (Qiagen). FISH probes were labeled by nick end translation using the FISH Tag DNA Kit for Alexa Fluor 555 or Alexa Fluor 488 (Invitrogen). 100 ng of each PCR product was combined and used to make a single FISH probe. SG4 cells were plated on poly-L-lysine coated slides and incubated with the fluorescent tagged probes exactly as described in [15]. Salivary gland FISH was performed as described in [65] and the protocol can be found online at http://www.igh.cnrs.fr/equip/cavalli/Lab%20Protocols/FISH-Immuno_Grimaud.pdf. Z-stacks were obtained using a Leica TCS-SP2 Spectral Laser Scanning Confocal Microscope (Leica Microsystems, GmbH, Wetzlar, Germany). Maximum projections of each image were made using Leica Confocal Software (Leica Microsystems). Quantification of the numbers of FISH probe signals per cell and the distances between signals in each cell were performed by manually scanning up and down through the 3-D projections of each Z stack and using the “Measurements” feature in the Volocity (Perkin Elmer) software.

FISH primers used for the control gene region 28B were:

28B F1 GAGTGACTTTGATCACAATCAGC

28B R1 CACATACGCACCGTTGGCC

28B F2 GGCCAACGGTGCGTATGTG

28B R2 GCTTTTGTGGGCAATGC

28B F3 GCATTGCCCACAAAAGC

28B R3 GATACCTCTGAAAGCAAAG

28B F4 CTTTGCTTTCAGAGGTATC

28B R4 GCTTTCGTTGCATCAGCAAGTC

28B F5 GACTTGCTGATGCAACGAAAGC

28B R5 GTGTCTTGAAAGTAGAAGGCAG

28B F6 CTGCCTTCTACTTTCAAGACAC

28B R6 CTAAGCCACTCACCCACAATC

28B F7 GATTGTGGGTGAGTGGCTTAG

28B R7 GCTCAATACCGCAACAGCCG

28B F8 CGGCTGTTGCGGTATTGAGC

28B R8 GAATCGGCAAATTCCAGCAC

28B F9 GTGCTGGAATTTGCCGATTC

28B R9 CAAACGCAATGAGCTTGGAC

28B F10 GTCCAAGCTCATTGCGAAAC

28B R10 CAGCACTCTCCGCACTTTGC

28B F11 GCAAAGTGCGGAGAGTGCTG

28B R11 GTTTGCCTTTCCTGCCACTCG

FISH primers used to amplify the region upstream of mdg1-1403 were:

mdg1-1403 FISH F1 GTTGGCTGGAACGCCCAGGATAC

mdg1-1403 FISH R1 GAATCTCCGACTCCGGACTTGTC

mdg1-1403 FISH F2 GACAAGTCCGGAGTCGGAGATTC

mdg1-1403 FISH R2 GGTCACATTGGTATCCCTCTCC

mdg1-1403 FISH F3 GGAGAGGGATACCAATGTGACC

mdg1-1403 FISH R3 GCCAGAATAGGTGGTAAGATCG

mdg1-1403 FISH F4 CGATCTTACCACCTATTCTGGC

mdg1-1403 FISH R4 CCTCGTATTTCTGAGTGACCAGTG

mdg1-1403 FISH F5 CACTGGTCACTCAGAAATACGAGG

mdg1-1403 FISH R5 CCTGACTGTTGCCAACAGTTAC

mdg1-1403 FISH F6 GTAACTGTTGGCAACAGTCAGG

mdg1-1403 FISH R6 CTTGTACACGTCCGAGAAAATACC

mdg1-1403 FISH F7 GGTATTTTCTCGGACGTGTACAAG

mdg1-1403 FISH R7 CGCATCGCTAGTACGTGTCTAG

mdg1-1403 FISH F8 CTAGACACGTACTAGCGATGCG

mdg1-1403 FISH R8 CGCCGATTATAAAACTGTATCCACC

mdg1-1403 FISH F9 GGTGGATACAGTTTTATAATCGGCG

mdg1-1403 FISH R9 CTTCAGGCCGTTGCAGTACACCTG

mdg1-1403 FISH F10 CAGGTGTACTGCAACGGCCTGAAG

mdg1-1403 FISH R10 GTTGGAAAACGGTGTTAGTCAGG

mdg1-1403 FISH F11 CCTGACTAACACCGTTTTCCAAC

mdg1-1403 FISH R11 GCTGATGGCATTGTAGCTTGG

mdg1-1403 FISH F12 CCAAGCTACAATGCCATCAGC

mdg1-1403 FISH R12 GTGCACTGACCTTGATCTGATTG

mdg1-1403 FISH F13 CAATCAGATCAAGGTCAGTGCAC

mdg1-1403 FISH F14 CCAACTTCGCTTGGTTGGAAG

FISH primers used to amplify the multi-copy mdg1 retrotransposon sequence were:

mdg1-1403 LTR F1 TCCTGTAGTTAATTAGAATTCCAATACTTCTG

mdg1-1403 LTR R1 CAAAAGGAGGGAGATGTAG

mdg1-1403 FISH F1 GTCTCAAAACGCAgttggtc

mdg1-1403 FISH R1 CAACACAACACCATCGGTAG

mdg1-1403 FISH F2 ctaccgatggtgttgtgttg

mdg1-1403 FISH R2 GACAGAAAAATACCTGCGCAGGTG

mdg1-1403 FISH F3 cacctgcgcaggtatttttctgtc

mdg1-1403 FISH R3 GGAGCATACCGCTACACGCGATTACC

mdg1-1403 FISH F4 ggtaatcgcgtgtagcggtatgctcc

mdg1-1403 FISH R4 CAAGGGACAATTCAGTCTCTAGG

mdg1-1403 FISH F5 cctagagactgaattgtcccttg

mdg1-1403 FISH R5 CAAAATGACAGACTCTGCCGCAAC

mdg1-1403 FISH F6 gttgcggcagagtctgtcattttg

mdg1-1403 FISH R6 GCCCGTAAAGCCATACACCAAC

mdg1-1403 FISH F7 gttggtgtatggctttacgggc

mdg1-1403 FISH R7 CTTAGGACCACCCTAATTCC

FISH primers used to amplify the region upstream of G2-1077 were:

G2-1077 FISH F1 CCCACCACTTTATCCTTGTAG

G2-1077 FISH R1 GAAGACATCAGCCGAAATGCG

G2-1077 FISH F2 CGCATTTCGGCTGATGTCTTC

G2-1077 FISH R2 GTGCCAGCTGTGTAAAGTCAGC

G2-1077 FISH F3 GCTGACTTTACACAGCTGGCAC

G2-1077 FISH R3 CCCTGGCGTCGTGCTCGACGAG

G2-1077 FISH F4 CTCGTCGAGCACGACGCCAGGG

G2-1077 FISH R4 GCAGTTGAACATCAGCATAAGG

G2-1077 FISH F5 CCTTATGCTGATGTTCAACTGC

G2-1077 FISH R5 GAGAACGTGCCGTGCCAAC

G2-1077 FISH F6 GTTGGCACGGCACGTTCTC

G2-1077 FISH R6 CAGAGCTTGTCTGCATATACAG

G2-1077 FISH F7 CTGTATATGCAGACAAGCTCTG

G2-1077 FISH R7 GGATGGTATTTACGGGAGGC

G2-1077 FISH F8 GCCTCCCGTAAATACCATCC

G2-1077 FISH R8 TCAAAAGTCCCGAGAAGTG

G2-1077 FISH F9 CACTTCTCGGGACTTTTGA

G2-1077 FISH R9 GAGATGTGGTCTCTTGGGTTG

G2-1077 FISH F10 CAACCCAAGAGACCACATCTC

G2-1077 FISH R10 GTTGCAATCCTTCTCGCGC

G2-1077 FISH F11 GCGCGAGAAGGATTGCAAC

G2-1077 FISH R11 CATTCGGTTGAACGTAGGGAC

G2-1077 FISH F12 GTCCCTACGTTCAACCGAATG

G2-1077 FISH R12 GAGCATCGAGCAGCAGGAGC

### Measurement of nuclear volumes

Volocity software (Perkin Elmer) was used to quantitate nuclear volumes in SG4 cells treated with control or dCAP-D3 dsRNA. Briefly, Z-stacks of DAPI stained nuclei were compressed into a single 3-D image and the “Population” tool in the “Measurement” feature was used to recognize the entire population of DAPI stained cells. The program was set to discard signal at the edges of the image in order to discard partial images of cells. Mitotic cells were also discarded from the measurements by individual selection. Finally, the list of measurements was exported to Excel where averaging and statistical analysis was performed.

### Chromatin immunoprecipitation

4×10∧7 cells per IP were used in all ChIP experiments. Cells were washed with PBS and then resuspended in 500 µL of buffer A (60 mM KCl, 15 mM NaCl, 4 mM MgCl2, 15 mM HEPES (pH 7.6), .5% Triton X-100, .5 mM DTT, EDTA-free protease inhibitors cocktail (Roche)) containing 1.8% formaldehyde. Resuspended cells were mixed for 15 minutes at RT. Glycine was added to a concentration of 225 mM and incubated at RT for 5 minutes. Samples were centrifuged at 4°C for 5 min at 4000 g. Supernatant was discarded and pellets were washed with 3 mL of buffer A. Samples were centrifuged as described above, supernatant was discarded, and pellets were resuspended in 500 µL of Hypertonic Buffer A (300 mM sucrose, 2 mM MgAcetate, 3 mM CaCl2, 10 mM Tris (pH 8.0), 0.1%Triton X-100, 0.5 mM DTT added fresh) and incubated at 4°C for 30 min with nutation. Samples were dounce homogenized 5× with a 2 mL homogenizer and tight pestle. Nuclei was collected by centrifuging for 5 min at 720 g at 4°C. Pellets were washed with 500 µL of Hypertonic Buffer A, centrifuged and resuspended in 500 µL of buffer D (25% glycerol, 5 mM MgAcetate, 50 mM tris (pH 8.0), 0.1 mM EDTA, 0.5 mM DTT added fresh). Samples were again centrifuged for 5 min at 720 g and then washed with 500 µL of buffer D. Samples were resuspended in 250 µL of buffer MN (60 mM KCl, 15 mM NaCl, 15 mM tris (pH 7.4), 0.5 mM DTT added fresh, .25M sucrose, 1 mM CaCl2 added fresh). 10 units of Micrococcal Nuclease (USB) were added to each sample and samples were incubated for 1 hour at RT. Reactions were stopped by adding 12.5 mM EDTA and .5% SDS. 10 µL of Dynal Protein A or G beads (Invitrogen) were used per µg of antibody and beads were prepared according to the manufacturer's recommendations. Beads were incubated with species specific IgG antibody, dCAP-D3 antibody (YZ384), γ-H2AV antibody (Rockland), H3K4me3 antibody (Abcam) or H3K9me3 antibody (Abcam) for 4 hours at RT with rotation. Beads were washed twice with 1 mL of .5% BSA/PBS solution and added to the diluted chromatin samples which were then incubated at 4°C overnight, with rotation. Samples were washed three times with wash buffer B (50 mM HEPES, pH 7.5, 100 mM LiCl, 1 mM EDTA, 1% NP-40, .1% Na-deoxycholate) and once with TE, with 5 minute rotation at 4°C in between each wash. TE was removed and bound protein was eluted by adding 202 µL of Elution Buffer (1%SDS, 10 mM EDTA, 50 mM Tris-Cl pH 8, mM NaCl) to each sample. Samples were incubated for 30 min at 65°C, with shaking at 500 rpm. Supernatants were transferred to new eppendorf tubes and incubated 6–16 hours at 65°C. 200 µL TE was added and samples were digested with Proteinase K and RNase A (Sigma), phenol-chloroform extracted, and ethanol precipitated. DNA pellets were dissolved in 105 µL of ddH2O and 3 µL was used per qRT-PCR reaction.

ChIP primers used:

*mdg1-1403* ChIP primers

ChIP primer set 1 = 5′gcaatggagaactggggtctgttg3′ AND 5′catgtgcgcctgttcgtgagc3′

ChIP primer set 2 = 5′caccgagcaggttggttatccc3′ AND 5′cagtgtagcattactgccatcgtc3′

ChIP primer set 3 = 5′gaataccggttgagaaccgtgctc3′ AND 5′ggcacgtactccacctccttc3′

ChIP primer set 4 = 5′ gctgcccgacttccggatatatc3′ AND 5′ gaccaactgcgttttgagac3′

ChIP primer set “LTR” = 5′ccaatgggagtcgagtgcgac3′ AND 5′ggaccaccctaattccttagggtc3′

ChIP primer set 5 = 5′gaccattggggtggtggagtg3′ AND 5′gcgatctgagtgagtagagtgtcag3′

ChIP primer set 6 = 5′caaatggctgtgcagataccaggc3′ AND 5′ccggcgatggtacttcatgacc3′

ChIP primer set 7 = 5′cagctgcacgagagactacgaaac3′ AND 5′gcctgaaccaagtcaggattctcc3′

ChIP primer set 8 = 5′cacggccgaggtgatcaatgac3′ AND 5′gacttggagagcagctcttccg3′

*G2-1077* ChIP primers

ChIP primer set 1 = 5′ctcggccctaaattgtccgttcg3′ AND 5′ctgctagctaatccgcgcttctc3′

ChIP primer set 2 = 5′cgaatgtctgcccactgcccac3′ AND 5′caatatgcagtggcacgagggtg3′

ChIP primer set 3 = 5′cggagttaatgaacctcctggcc3′ AND 5′cataggtggctgctgtgaggtaac3′

ChIP primer set 4 = 5′cgcctaaagcaactccactggc3′ AND 5′gcttgcagtgccacacagctg3′

ChIP primer set 5 = 5′ gtgttggatgtcaagctcaactgac3′ AND 5′caagaagacaaacagattttggcacgc

ChIP primer set 6 = 5′ gtgatgtcagttggcacagttggc3′ AND 5′ggcgtgaacacatttagaaggaactcc

## Supporting Information

Figure S1 dCAP-D2 knockdown in SG4 cells does not result in a local loss of retrotransposon sequence. A) qRT-PCR for dCAP-D2 transcript levels shows a significant decrease in SG4 cells treated with dCAP-D2 dsRNAs after 4 days of treatment (dark grey bar) in comparison to cells treated with control dsRNA (black bar). (*) indicates p-value less than 0.05 as calculated by student unpaired t-test. PCR for B) *mdg1-1403* and C) *G2-1077* presence or absence in SG4 cells treated with dsRNAs targeting dCAP-D2 indicate only presence of retrotransposon sequence. PCRs were performed on cells treated with 1) control dsRNA to test for presence, 2) control dsRNA to test for absence, 3) dCAP-D2 dsRNA to test for presence and 4) dCAP-D2 dsRNA to test for absence. Tubulin23C (Tub) was used as a control for each reaction. In the PCRs performed on the *mdg1-1403* locus, an asterisk denotes the band for presence. The miscellaneous band seen in the wild type absence reaction was confirmed to be a mispriming event off of tubulin (data not shown).
(TIF)

Figure S2 Time course of dCAP-D3 knockdown in SG4 cells indicates local loss of retrotransposon sequence occurs the day after the greatest decrease in dCAP-D3 levels. A) qRT-PCR for *dCap-D3* transcript levels over a 6 day time course demonstrates that the greatest decrease in dCAP-D3 dsRNA treated SG4 cells (white bars) occurs on day 4, as compared to cells treated with control dsRNA (black bars). Transcript levels were normalized to housekeeping gene *rp49*. B) DNA was harvested from SG4 cells over the time course of dsRNA treatment described in A and PCRs were performed (as described in Figure 1) to check for presence (top) and absence (bottom) of *mdg-1403*. dCAP-D3 dsRNA treated cells (right) exhibit appearance of an absence band on day 5, but control dsRNA treated cells (left) do not. (*) indicates p-value less than 0.05, as calculated by student unpaired t-test. C) Sequencing of cloned “absence” PCR products (described in Figure 1) for *mdg1-1403*(top) and *X-978* (bottom) from *dCap-D3* mutant adults and SG4 cells treated with dCAP-D3 dsRNAs reveal the precise loss of retrotransposon sequence and the retention of one copy of a small repeated sequence normally found in two copies positioned immediately before and after the retrotransposon sequence. Cloning vector sequence is shown in blue, upstream neighboring DNA sequence in yellow, downstream neighboring DNA sequence in pink, and the small repeat sequences are shown in green. Representative sequences of 5 experiments per retrotransposon from SG4 cells are shown.
(TIF)

Figure S3 Knockdown of Dicer2 in SG4 cells increases retrotransposon transcripts but does not result in a local loss of retrotransposon sequence. A) qRT-PCR demonstrates a significant decrease *dicer2* transcripts in SG4 cells treated with DICER2 dsRNAs after 4 days of treatment (dark grey bar) in comparison to cells treated with control dsRNA (black bar). DICER2 knockdown results in 2 fold increases in transcript levels of mdg1 and G2 transcripts but no change in X element transcripts. (*) indicates p-value less than 0.05 as calculated by student unpaired t-test. PCR for B) *mdg1-1403* and C) *G2-1077* presence or absence in SG4 cells treated with dsRNAs targeting Dicer2 indicate only presence of retrotransposon sequence. PCRs were performed on cells treated with 1) control dsRNA to test for presence, 2) control dsRNA to test for absence, 3) DICER2 dsRNA to test for presence and 4) DICER2 dsRNA to test for absence. Tubulin23C (Tub) was used as a control for each reaction. In the PCRs performed on the *mdg1-1403* locus, an asterisk denotes the band for presence. The miscellaneous band seen in the wild type absence reaction was confirmed to be a mispriming event off of tubulin (data not shown).
(TIF)

Figure S4 Decreased dCAP-D3 expression does not affect copy number of single copy, non-retrotransposon genes. Copy numbers of two single copy genes, A) CG31198 and B) CG32440, located immediately upstream of the *mdg1-1403* or *G2-1077* retrotransposons, respectively, were measured in wild type (black bars) and *dCap-D3* mutant (white bars) larvae. Copy numbers for each gene were normalized to each other.
(TIF)

Figure S5 dCAP-D3 knockdown in SG4 cells has no dramatic effect on the cell cycle distribution. SG4 cells were treated with Control (T7) dsRNAs or dCAP-D3 dsRNAs for 4, 5, or 6 days, stained with propidium iodide and analyzed by FACS. Results shown are representative of two independent experiments and demonstrate the cell cycle profile does not change by more than 1.5% on any given day.
(TIF)

Figure S6 Double strand breaks accumulate within the G2 retrotransposon sequence following dCAP-D3 dsRNA expression. ChIP for γ-H2AV performed on the *G2-1077* locus in SG4 cells treated with control dsRNA (black bars) demonstrates higher levels of binding in the region which flanks the retrotransposon sequence. ChIP in cells treated with dCAP-D3 dsRNA (white bars) show a shift in γ-H2AV distribution out of retrotransposon flanking regions and into retrotransposon sequence. Primer sets used are depicted above the charts. Primer set “4” is not specific for the locus but instead primes global retrotransposon sequence. Results are the averages of 2 experiments involving duplicate IPs and are presented as a percentage of the IP with control IgG ChIP signal subtracted. (*) and (**) indicate quantitative comparisons between IgG signal and dCAP-D3 signal with a p-value less than 0.05 or 0.01, respectively, as calculated by student unpaired t-test. (+) indicates a quantitative comparison of specific dCAP-D3 signal to the average over the entire locus with a p-value less than 0.05 as calculated by student unpaired t-test.
(TIF)

Figure S7 *dCap-D3* mutant salivary glands exhibit loss of *mdg1-1403* and increases in retrotransposon copy number at other loci. FISH experiments using probes hybridized to the *mdg1-1403* locus (green) and to *mdg1* retrotransposon sequence (red) demonstrate that wild type glands retain the *mdg1* retrotransposon sequence at the *mdg1-1403* locus (middle panel and smaller panels on right in A) while *dCap-D3* mutants do not (middle panel and smaller panels on right in B). Yellow arrows indicate co-localization of probes in wild type preparations and absence of co-localization in *dCap-D3* mutant preparations. The average copy number for each genotype was determined for salivary glands from 5 separate larvae by counting total numbers of *mdg1* bands. Salivary gland chromatin was stained with DAPI and is shown in white.
(TIF)
