# Supplementary figures and images for: Condensin II Subunit dCAP-D3 Restricts Retrotransposon Mobilization in Drosophila Somatic Cells
Source: PLoS Genet. 2013 Oct 31;9(10):e1003879. doi: 10.1371/journal.pgen.1003879 (PMC3814330; doi:10.1371/journal.pgen.1003879)

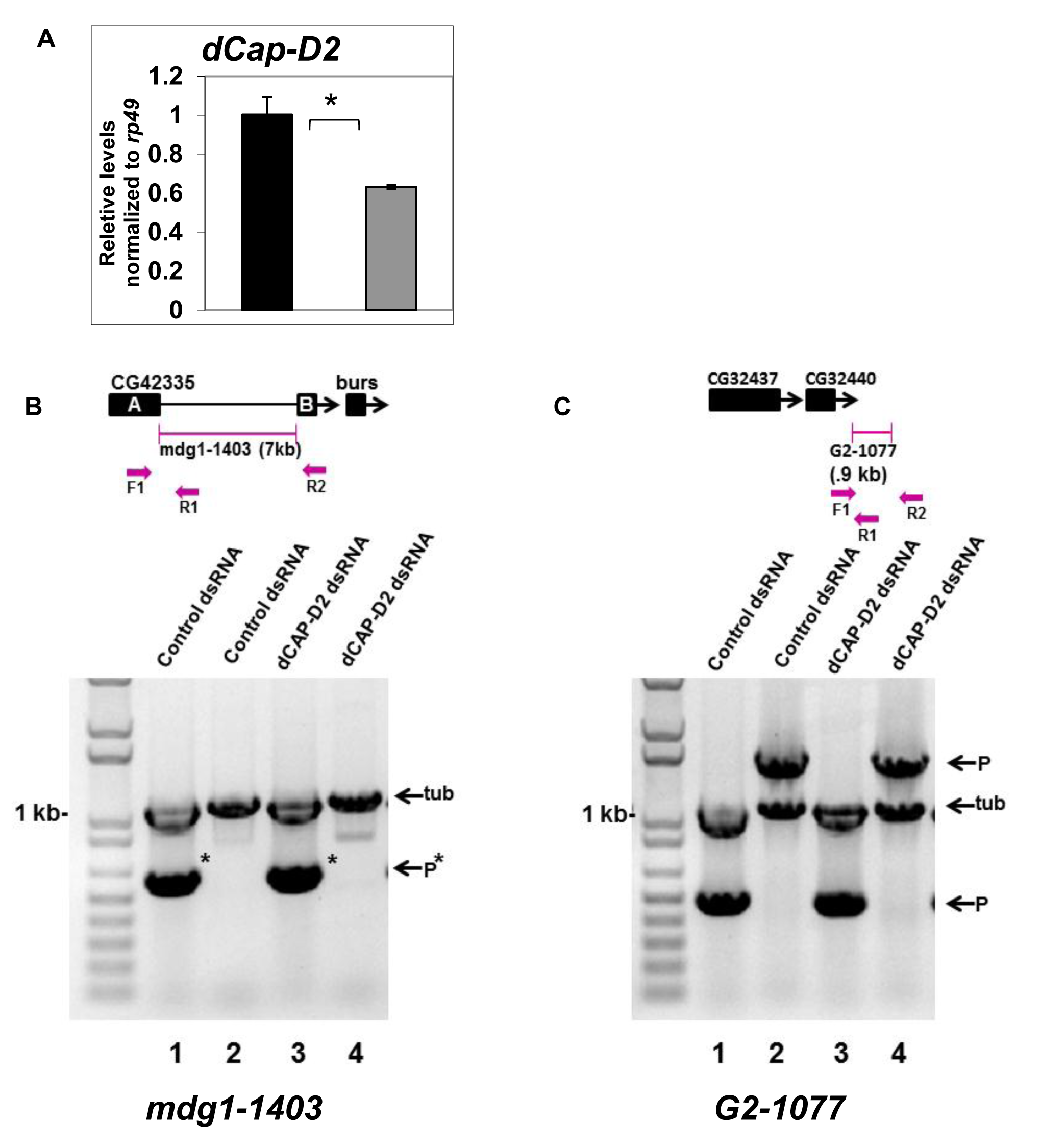

Supplement: Figure S1 — dCAP-D2 knockdown in SG4 cells does not result in a local loss of retrotransposon sequence. A) qRT-PCR for dCAP-D2 transcript levels shows a significant decrease in SG4 cells treated with dCAP-D2 dsRNAs after 4 days of treatment (dark grey bar) in comparison to cells treated with control dsRNA (black bar). (*) indicates p-value less than 0.05 as calculated by student unpaired t-test. PCR for B) mdg1-1403 and C) G2-1077 presence or absence in SG4 cells treated with dsRNAs targeting dCAP-D2 indicate only presence of retrotransposon sequence. PCRs were performed on cells treated with 1) control dsRNA to test for presence, 2) control dsRNA to test for absence, 3) dCAP-D2 dsRNA to test for presence and 4) dCAP-D2 dsRNA to test for absence. Tubulin23C (Tub) was used as a control for each reaction. In the PCRs performed on the mdg1-1403 locus, an asterisk denotes the band for presence. The miscellaneous band seen in the wild type absence reaction was confirmed to be a mispriming event off of tubulin (data not shown). (TIF) [file pgen.1003879.s001.tif]

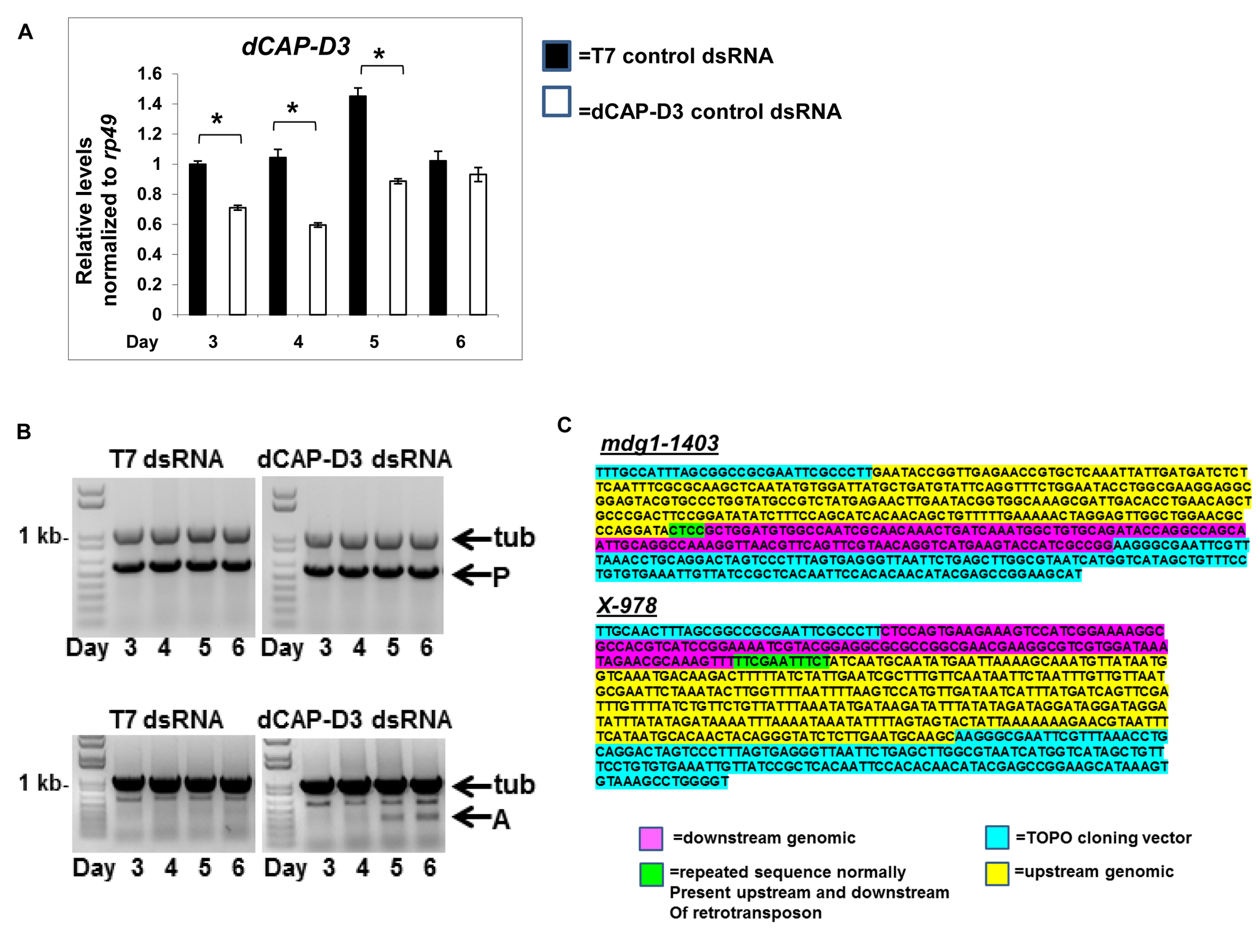

Supplement: Figure S2 — Time course of dCAP-D3 knockdown in SG4 cells indicates local loss of retrotransposon sequence occurs the day after the greatest decrease in dCAP-D3 levels. A) qRT-PCR for dCap-D3 transcript levels over a 6 day time course demonstrates that the greatest decrease in dCAP-D3 dsRNA treated SG4 cells (white bars) occurs on day 4, as compared to cells treated with control dsRNA (black bars). Transcript levels were normalized to housekeeping gene rp49. B) DNA was harvested from SG4 cells over the time course of dsRNA treatment described in A and PCRs were performed (as described in Figure 1) to check for presence (top) and absence (bottom) of mdg-1403. dCAP-D3 dsRNA treated cells (right) exhibit appearance of an absence band on day 5, but control dsRNA treated cells (left) do not. (*) indicates p-value less than 0.05, as calculated by student unpaired t-test. C) Sequencing of cloned “absence” PCR products (described in Figure 1) for mdg1-1403(top) and X-978 (bottom) from dCap-D3 mutant adults and SG4 cells treated with dCAP-D3 dsRNAs reveal the precise loss of retrotransposon sequence and the retention of one copy of a small repeated sequence normally found in two copies positioned immediately before and after the retrotransposon sequence. Cloning vector sequence is shown in blue, upstream neighboring DNA sequence in yellow, downstream neighboring DNA sequence in pink, and the small repeat sequences are shown in green. Representative sequences of 5 experiments per retrotransposon from SG4 cells are shown. (TIF) [file pgen.1003879.s002.tif]

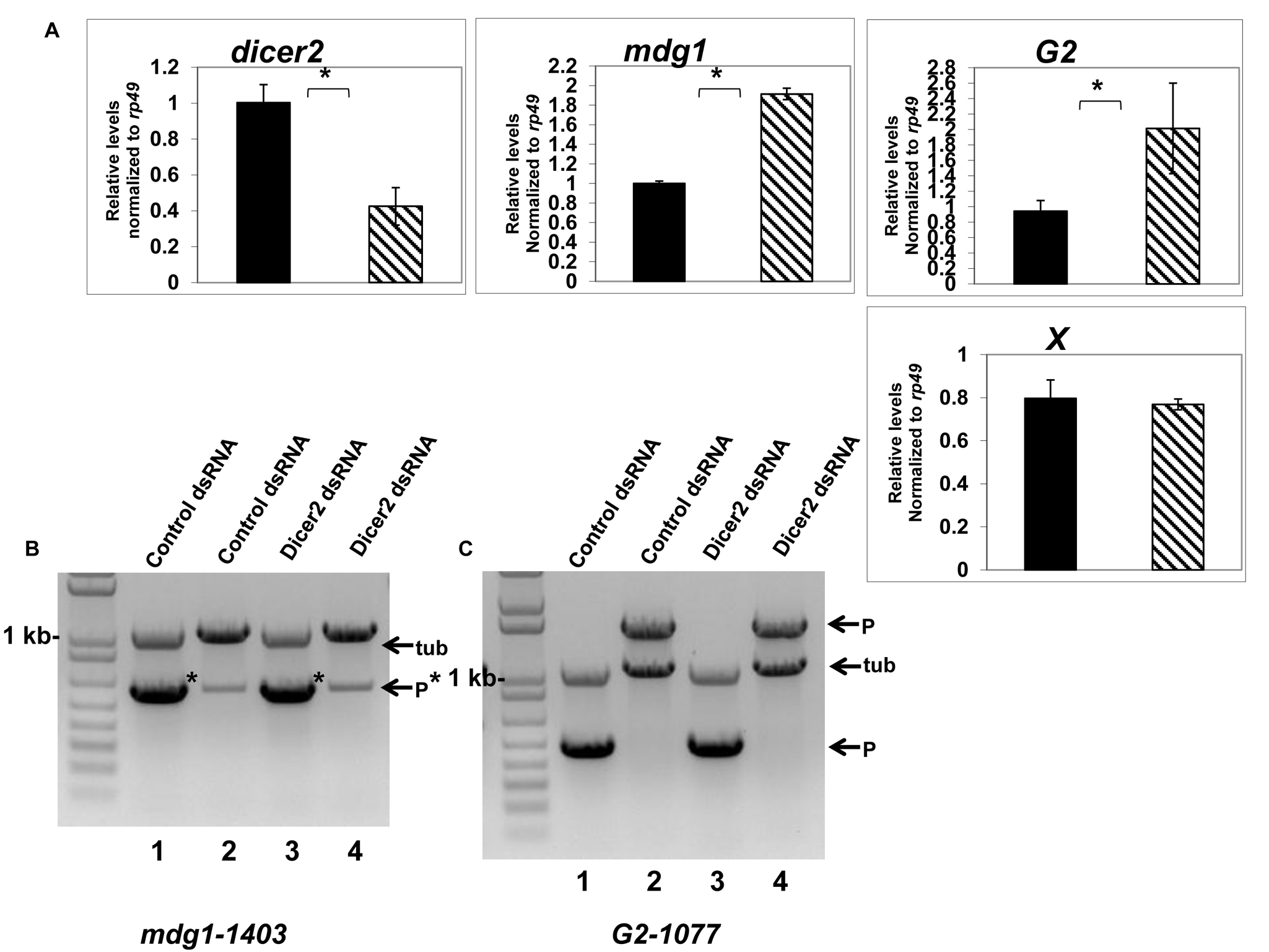

Supplement: Figure S3 — Knockdown of Dicer2 in SG4 cells increases retrotransposon transcripts but does not result in a local loss of retrotransposon sequence. A) qRT-PCR demonstrates a significant decrease dicer2 transcripts in SG4 cells treated with DICER2 dsRNAs after 4 days of treatment (dark grey bar) in comparison to cells treated with control dsRNA (black bar). DICER2 knockdown results in 2 fold increases in transcript levels of mdg1 and G2 transcripts but no change in X element transcripts. (*) indicates p-value less than 0.05 as calculated by student unpaired t-test. PCR for B) mdg1-1403 and C) G2-1077 presence or absence in SG4 cells treated with dsRNAs targeting Dicer2 indicate only presence of retrotransposon sequence. PCRs were performed on cells treated with 1) control dsRNA to test for presence, 2) control dsRNA to test for absence, 3) DICER2 dsRNA to test for presence and 4) DICER2 dsRNA to test for absence. Tubulin23C (Tub) was used as a control for each reaction. In the PCRs performed on the mdg1-1403 locus, an asterisk denotes the band for presence. The miscellaneous band seen in the wild type absence reaction was confirmed to be a mispriming event off of tubulin (data not shown). (TIF) [file pgen.1003879.s003.tif]

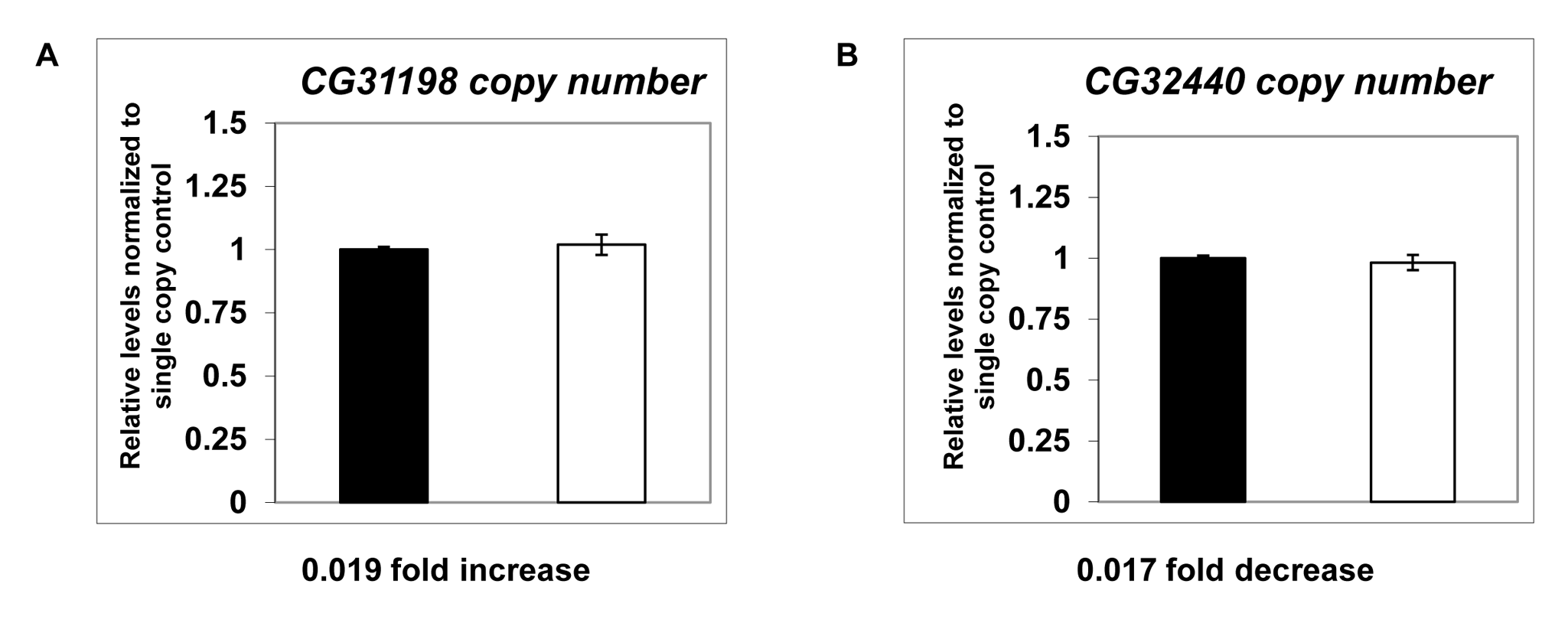

Supplement: Figure S4 — Decreased dCAP-D3 expression does not affect copy number of single copy, non-retrotransposon genes. Copy numbers of two single copy genes, A) CG31198 and B) CG32440, located immediately upstream of the mdg1-1403 or G2-1077 retrotransposons, respectively, were measured in wild type (black bars) and dCap-D3 mutant (white bars) larvae. Copy numbers for each gene were normalized to each other. (TIF) [file pgen.1003879.s004.tif]

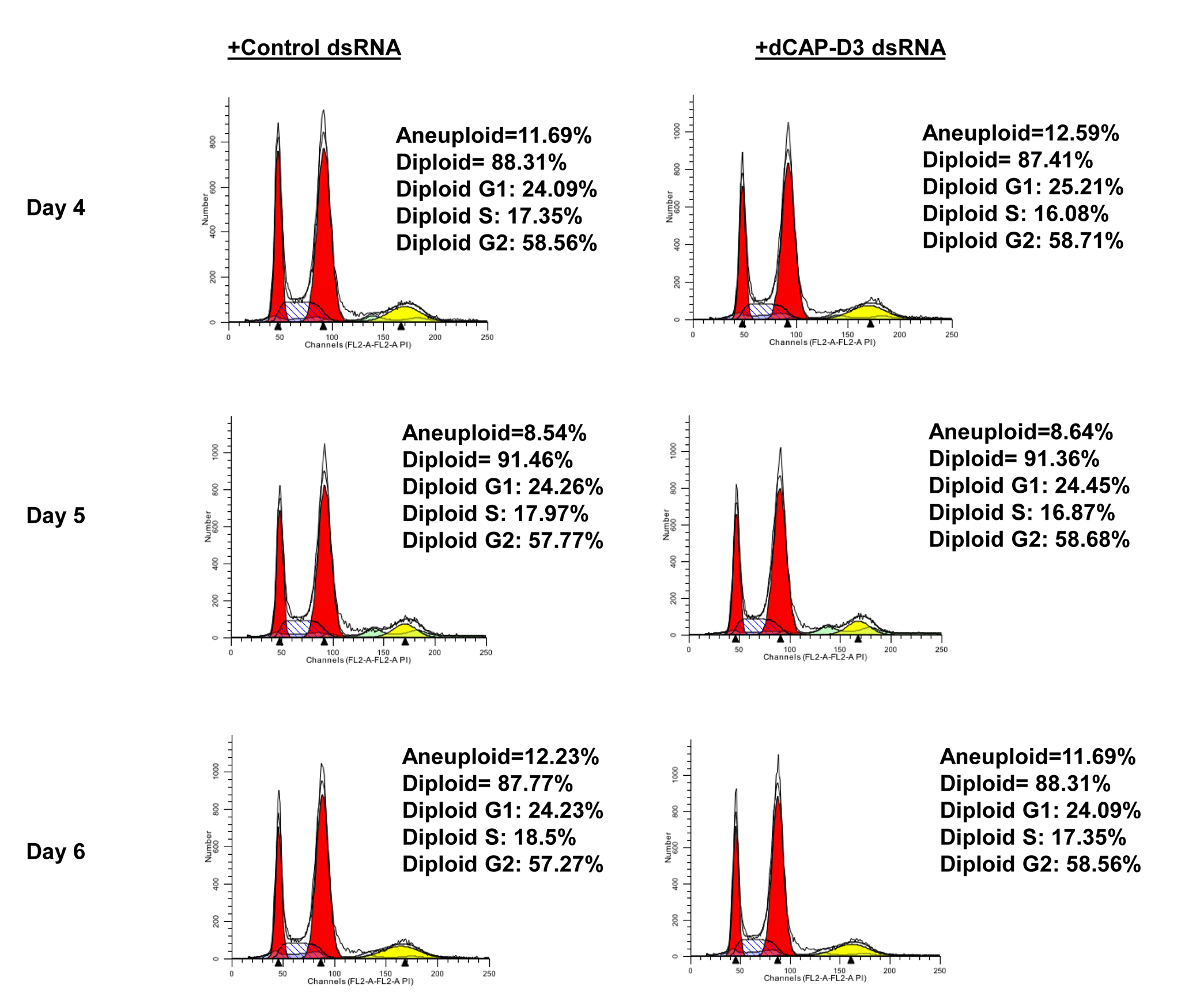

Supplement: Figure S5 — dCAP-D3 knockdown in SG4 cells has no dramatic effect on the cell cycle distribution. SG4 cells were treated with Control (T7) dsRNAs or dCAP-D3 dsRNAs for 4, 5, or 6 days, stained with propidium iodide and analyzed by FACS. Results shown are representative of two independent experiments and demonstrate the cell cycle profile does not change by more than 1.5% on any given day. (TIF) [file pgen.1003879.s005.tif]

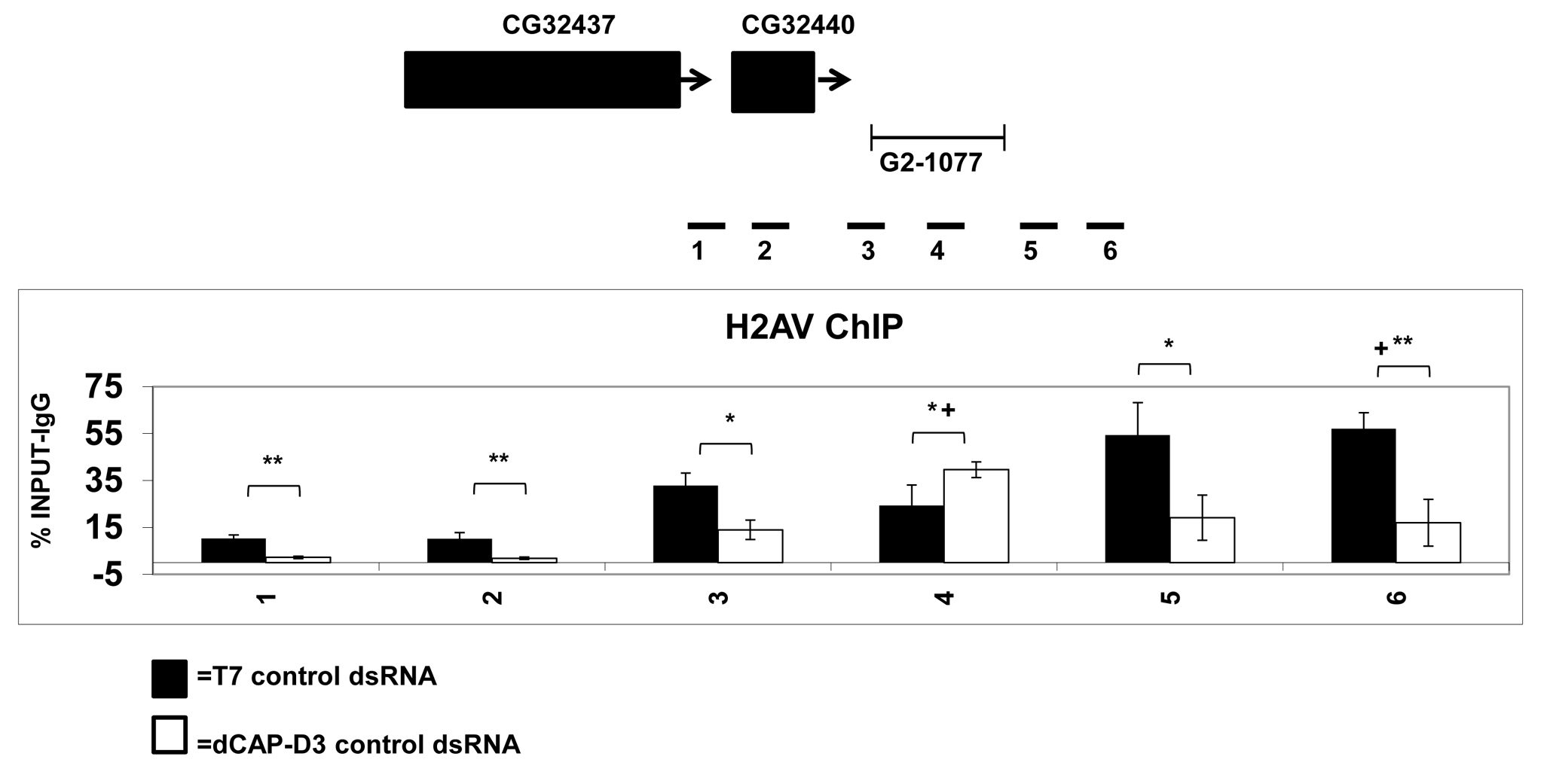

Supplement: Figure S6 — Double strand breaks accumulate within the G2 retrotransposon sequence following dCAP-D3 dsRNA expression. ChIP for γ-H2AV performed on the G2-1077 locus in SG4 cells treated with control dsRNA (black bars) demonstrates higher levels of binding in the region which flanks the retrotransposon sequence. ChIP in cells treated with dCAP-D3 dsRNA (white bars) show a shift in γ-H2AV distribution out of retrotransposon flanking regions and into retrotransposon sequence. Primer sets used are depicted above the charts. Primer set “4” is not specific for the locus but instead primes global retrotransposon sequence. Results are the averages of 2 experiments involving duplicate IPs and are presented as a percentage of the IP with control IgG ChIP signal subtracted. (*) and (**) indicate quantitative comparisons between IgG signal and dCAP-D3 signal with a p-value less than 0.05 or 0.01, respectively, as calculated by student unpaired t-test. (+) indicates a quantitative comparison of specific dCAP-D3 signal to the average over the entire locus with a p-value less than 0.05 as calculated by student unpaired t-test. (TIF) [file pgen.1003879.s006.tif]

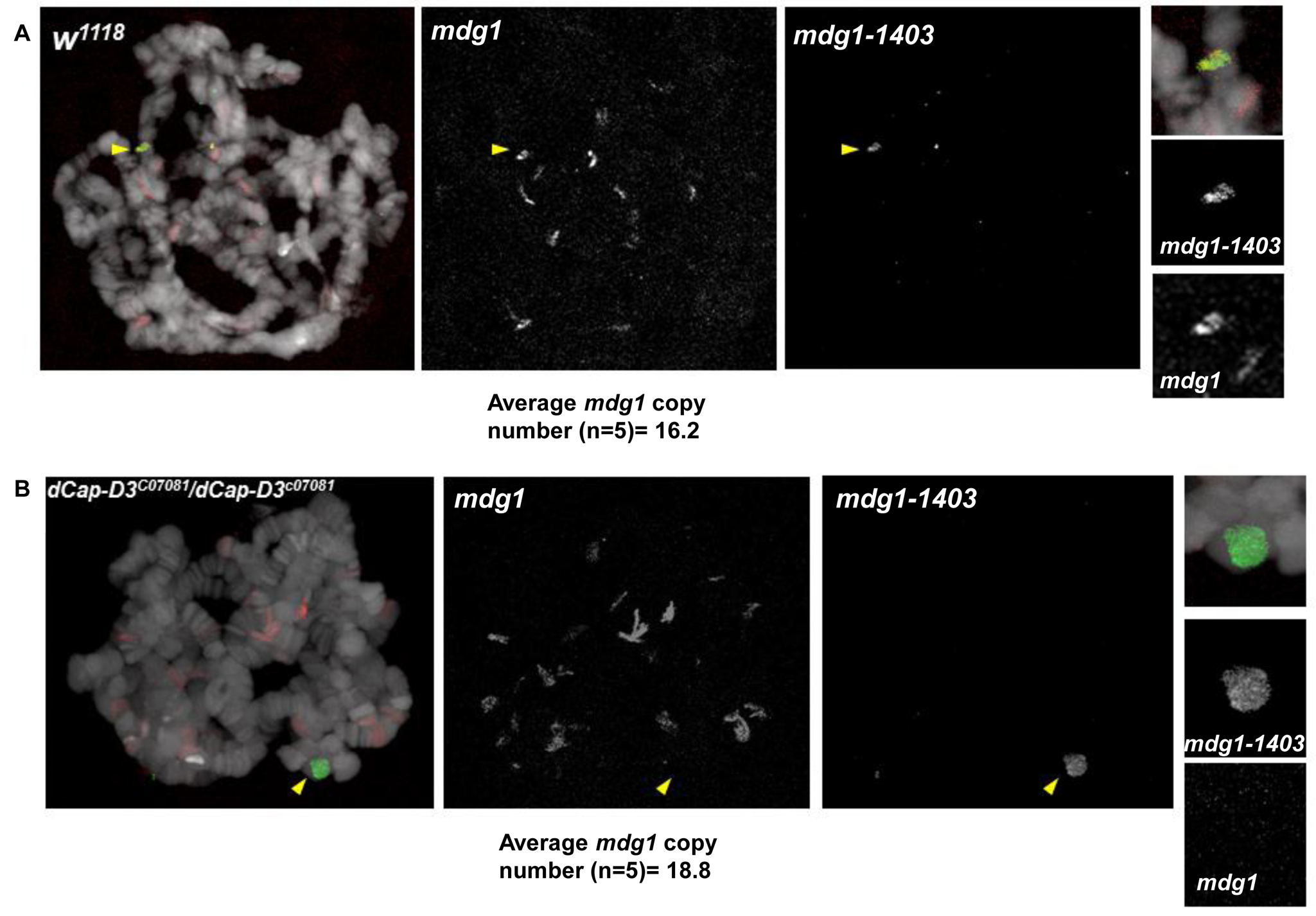

Supplement: Figure S7 — dCap-D3 mutant salivary glands exhibit loss of mdg1-1403 and increases in retrotransposon copy number at other loci. FISH experiments using probes hybridized to the mdg1-1403 locus (green) and to mdg1 retrotransposon sequence (red) demonstrate that wild type glands retain the mdg1 retrotransposon sequence at the mdg1-1403 locus (middle panel and smaller panels on right in A) while dCap-D3 mutants do not (middle panel and smaller panels on right in B). Yellow arrows indicate co-localization of probes in wild type preparations and absence of co-localization in dCap-D3 mutant preparations. The average copy number for each genotype was determined for salivary glands from 5 separate larvae by counting total numbers of mdg1 bands. Salivary gland chromatin was stained with DAPI and is shown in white. (TIF) [file pgen.1003879.s007.tif]
